# Effectiveness of guided telerehabilitation on functional performance in community-dwelling older adults: A systematic review

**DOI:** 10.1177/02692155231217411

**Published:** 2023-11-28

**Authors:** CJ Gamble, JCM van Haastregt, EF van Dam van Isselt, SMG Zwakhalen, JMGA Schols

**Affiliations:** 1 Department of Health Services Research, Faculty of Health Medicine and Life Sciences, CAPHRI Care and Public Health Research Institute, Maastricht, The Netherlands; 2 Living Lab of Ageing and Long Term Care, Maastricht, The Netherlands; 3 Stichting Valkenhof, Valkenswaard, The Netherlands; 4 University Network for the Care sector Zuid-Holland, 4501 Leiden University Medical Centre, Leiden, The Netherlands; 5 Department of Public Health and Primary Care, 4501 Leiden University Medical Centre, Leiden, The Netherlands

**Keywords:** Telerehabilitation, effectiveness, older adults, functional performance

## Abstract

**Objective:**

To systematically review the effectiveness of guided telerehabilitation on improving functional performance in community-dwelling older adults.

**Data sources:**

Articles published in PubMed, Cochrane Library and Embase (Ovid) from 01 January 2010 up to 17 October 2023.

**Review Methods:**

Included studies had (1) a randomised controlled trial design, (2) an average population age of 65 years or older, (3) a home-based setting and (4) evaluated the effectiveness of functional performance outcome measures. The intervention was considered telerehabilitation when guided by a healthcare professional using video, audio and/or text communication technologies with a minimum frequency of once per week. The Preferred Reporting Items for Systematic Reviews and Meta-Analysis 2020 statement guideline was followed. Methodological quality was appraised using the revised Cochrane Risk of Bias tool.

**Results:**

A total of 26 randomised controlled trials were included. Telerehabilitation had superior (*N *= 15), non-superior (*N *= 16) or non-inferior (*N *= 11) effectiveness for improving functional performance outcome measures compared to control interventions. No studies found the control intervention to be superior over telerehabilitation. Between study differences in intervention characteristics contributed to significant clinical heterogeneity. Five studies were found to present an overall ‘low’ risk of bias, 12 studies to present ‘some’ risk of bias and 9 studies to present an overall ‘high’ risk of bias.

**Conclusion:**

The findings suggest that telerehabilitation could be a promising alternative to in-person rehabilitation for improving functional performance in community-dwelling older adults. Additional well-designed studies with minimised bias are needed for a better understanding of effective telerehabilitation intervention strategies.

## Introduction

Rehabilitation represents a core component of healthcare services provided for many conditions community-dwelling older adults may be affected by.^1,2^ Geriatric rehabilitation is defined as ‘*a multidimensional approach of diagnostic and therapeutic interventions, the purpose of which is to optimise functional capacity, promote activity and preserve functional reserve and social participation in older people with disabling impairments*’*.*^3^ Given the aging population, workforce shortages and increased demand for geriatric rehabilitation,^4^ healthcare systems are engaged in a process of innovation to improve efficiency.^5,6^

Recently, innovative information and communication technologies have provided convenient and affordable tools to extend healthcare services across geographic, time and economic barriers.^7^ Telerehabilitation, defined as ‘*the delivery of rehabilitation interventions to patients at a distance using information and communication technologies*’,^8^ allows for substituting the traditional in-person approach for rehabilitation at any place and any time, guided remotely by healthcare professionals. Over time, additional telerehabilitation advantages have emerged, such as the possibility to better integrate patient skills into daily life^9^ and to allow for efficient monitoring of rehabilitation progress.^10,11^

Despite these benefits, the lack of digital literacy, safety issues and age-related conditions could impair the use of telerehabilitation in an older adult population.^12,13^ However, higher age alone was found not to be an obstacle when using telerehabilitation.^14^ In fact, as early as 2006, the possible feasibility and effectiveness of home-based telerehabilitation in geriatric patients was already demonstrated.^15^ Moreover, global data shows that engagement with internet facilities among older adults has tripled to 67% since 2004.^16^ Additionally, safety issues have previously been successfully addressed in telerehabilitation research by involving patients’ partner or other informal caregivers to supervise exercises.^17–20^

A growing body of literature reviews support the effectiveness of telerehabilitation for improving health outcomes for neurological,^21–24^ oncological,^25^ musculoskeletal^9,26–28^ and cardiopulmonary conditions.^29–31^ However, only a limited number of literature reviews have more specifically targeted community-dwelling older adults.^7,32–35^ Evidence of these reviews suggest that telerehabilitation can achieve equal outcomes when compared to conventional rehabilitation methods. Yet, the generalisability of this evidence for community-dwelling older adults is limited, due to focussing specifically on the South-East Asia region,^32^ focussing on one specific telerehabilitation methodology,^34^ including middle-aged adults,^35^ not exclusively including home-based interventions^33,34^ or not following reporting guidelines.^7,33^

For older adults in need of rehabilitation, the main recommendation is to improve the outcome of functional performance.^3^ An older adult's functional performance is influenced by many factors, such as postural control and stability, lower extremity strength, dynamic balance and overall endurance.^36^ While there is no generally accepted definition of functional performance, the essence is ‘*the ability to safely and effectively perform functional tasks necessary for daily living*’.^36^ Currently, no previous review has primarily focused on the construct of functional performance as the outcome measure of interest. Therefore, the aim of this review is to (1) study the current evidence on the effectiveness of guided telerehabilitation on functional performance for community-dwelling older adults and (2) determine if telerehabilitation is non-inferior in results to conventional, in-person rehabilitation.

## Methods

This systematic review was reported according to the Preferred Reporting Items for Systematic Reviews and Meta-Analysis (PRISMA) statement.^37^ The protocol for this systematic review was registered with PROSPERO on 05 July 2020 with protocol ID CRD42020179231.

The main author (CJG) developed the search strategy which was reviewed by an experienced librarian of Maastricht University, as well as the co-authors (JCMH and JMGAS) for completeness. The following databases for studies published from 01 January 2010 up to 17 October 2023 were searched: Medline (PubMed), The Cochrane Library and Embase (Ovid). Only research published after 01 January 2010 was included due to significant and impactful developments made in communication technologies in recent years. Databases were first searched in April through May 2020, updated in January through April 2022, with final updates in May and October 2023. The search strategy included subject headings and keywords to capture the concepts of telerehabilitation, older adults and randomised controlled trial type publications. An adapted version of the high sensitivity randomised controlled trial search strategy was applied.^38^ The search strategy was adjusted to be appropriate for each included database. All search strategies can be found in the Supplemental files (page 2–4). For all studies accepted at the full text level, we conducted a forward citation search as well as reviewing reference lists to identify potential additional articles. Lastly, systematic reviews published since 01 January 2010 on a similar subject matter were reviewed for additional references. All identified citations were uploaded to EndNote (X9.3.3, Thomson Reuters).

### Study selection

All database search results were merged and uploaded to EndNote with duplicate records removed. The remaining papers were first screened on title and subsequently on abstract, performed by the main author (CJG). The remaining potentially relevant reports were then examined at the full text level by the main author (CJG) and one co-author (JCMH). The co-author (JMGAS) adjudicated for unclear decisions. Studies were included based on the established inclusion and exclusion criteria.

The following inclusion criteria were applied. Randomised controlled trial publications in the English language were included. The population of interest were community-dwelling older adults with an average age of 65 years or older. The study setting was home-based, independent living situations. Telerehabilitation was defined as ‘*the delivery of rehabilitation interventions to patients at a distance using information and communication technologies*’.^8^ For the purpose of this review, a broad interpretation of this definition was adopted to gain a comprehensive overview of methods of application. Thus, studies on telerehabilitation were included as ‘*any rehabilitation intervention aimed to optimise functional performance, promote activity and preserve functional reserve and social participation, that was guided by a healthcare professional by video, audio and/or text communication technologies*’. Guidance was defined as ‘*direct contact via information and communication technologies between the healthcare professional and patient*’. A minimum of 75% guidance by telerehabilitation was required for inclusion, to allow for occasional in-person visits. The method of information and communication technology chosen determined the guidance synchronicity of the study rehabilitation intervention. Guidance synchronicity refers to whether each study intervention session was guided real-time or not, where synchronous guidance (e.g. videoconferencing) is simultaneous to the study intervention and a-synchronous guidance (e.g. phone, app or web-based communication) is a-synchronous to the study intervention. Guidance frequency was set at a minimum of once per week, on the basis that treatment must be provided that aligns with clinical practise guidelines and evidence-based recommendations.^12,39^ The control intervention was considered to be usual care, an alternative form of in-person rehabilitation or no care. The control group could not be an alternative form of telerehabilitation. The outcome measure of interest was the construct of functional performance.^3^ An older adult's functional performance is influenced by many factors and cannot be captured in a single performance test.^36^ Therefore, several specific performance tests were pre-determined to assess the construct of functional performance^36^: 6-minute walking distance,^40–42^ Timed-Up-and–Go test,^43,44^ Berg Balance Scale,^45,46^ Shuttle Walk test,^40,42^ Sit-To-Stand test^47^ and 10m walk test.^48^ These tests have shown adequate reliability and validity and represent the multicomponent domains of cardiorespiratory endurance, balance, stability, muscular strength and functional mobility that contribute to functional performance.^36^

### Data extraction

The main author (CJG) was responsible for extracting the following information from the included studies using an adapted version of the Cochrane collaboration data collection form: author; publication year; country of origin; funding; conflict of interest; population (n), sex, age, primary medical condition; recruitment and retention; setting; intervention and control groups, intervention modality and details, frequency of guidance, frequency of use; adverse events and safety measures. The main author (CJG) and co-author (JCMH) were responsible for extracting the following information critical to the interpretation of results in order to minimise errors and reduce potential biases: study outcomes and main findings.

### Risk of bias assessment

The revised Cochrane Risk-of-Bias Tool for randomised studies was used to assess the methodological quality of included studies.^49^ By this tool, the risk of bias was assessed on the following domains: the randomisation process, deviations from the intended interventions, missing outcome data, measurement of the outcome and selection of the reported results. Each domain consists of several signalling questions for assigning one of the three levels of risk of bias judgement. The main author (CJG) was responsible for rating each domain as having ‘low risk’ of bias, ‘some concerns’ for bias or having a ‘high risk’ of bias, resulting in a corresponding overall score for each study. The co-authors (JCMH and JMGAS) adjudicated for unclear decisions.

## Results

**Figure 1.**
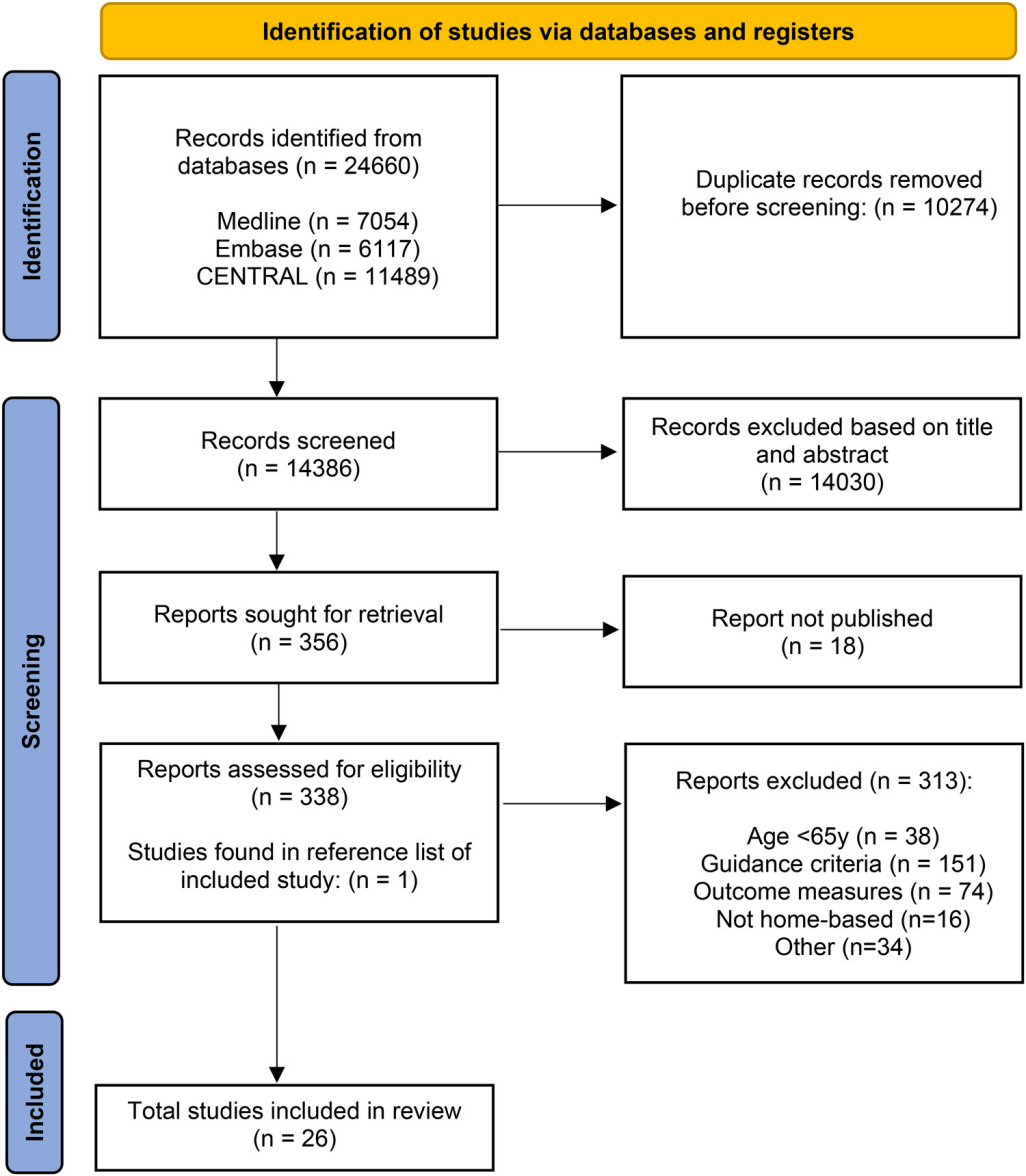
The PRISMA 2020 statement: an updated guideline for reporting systematic reviews flow diagram.

The search strategy identified 24,660 studies in total. After the removal of duplicates, 14,386 studies were screened on title and abstract, after which 14,030 studies were excluded. The remaining 356 studies were assessed full-text for eligibility. Based on this assessment, 331 studies were excluded with reasons including unavailable full text reports (n = 18), ineligible participant age (n = 38), ineligible guidance criteria (n = 151), ineligible outcome measures (n = 74), ineligible setting (n = 16) or other (n = 34). One study was identified from other sources, as described in the search strategy. A total of 26 eligible studies were identified and included in this review. The process is outlined in the flow diagram below (Figure 1).

### Study characteristics

The study characteristics of the 26 included studies are outlined in Table 1. Further study details can be found in the Supplemental files. The included studies originated from the United States (n = 4),^50–53^ South-Korea (n = 4),^54–57^ Australia (n = 3),^58–60^ Italy (n = 3),^18,61,62^ Canada (n = 3),^17,63,64^ China (n = 3),^19,20,65^ Turkey (n = 2),^66,67^ the United Kingdom (n = 1),^68^ Denmark (n = 1),^69^ Norway (n = 1)^70^ and Cyprus (n = 1).^71^ The number of participants in any single study varied from 20 to 306, with the lowest reported mean study age being 65.0 (± 8.0) years and the highest reported mean study age 83.0 (± 4.8) years. Among the 26 included studies, the following medical conditions were reported; chronic obstructive pulmonary disease (n = 5),^58,60,62,68,69^ total knee arthroplasty (n = 4),^17,50,56,63^ chronic heart failure (n = 4),^19,59,62,70^ total hip arthroplasty (n = 1),^64^ coronary artery disease (n = 1),^65^ stroke (n = 1),^20^ chronic lower back pain (n = 1),^52^ Parkinson's disease (n = 1),^18^ Alzheimer's disease (n = 1),^66^ COVID-19 (n = 1)^67^ and sarcopenia (n = 1).^54^ Six studies reported on community-dwelling older adults with increased fall risk,^51,53,55,57,61,71^ with no specific medical condition pre-defined.

**Table 1.** Study characteristics and main findings of included randomised controlled studies (n = 26).

	Telerehabilitation guidance characteristics		Study details				Population N, mean age ± SD	
Study	Guidance methodology	Guidance frequency	Study aim	Intervention duration	FP outcome measure(s)	Main findings ‡	Intervention	Control
**Alpozgen et al.** **^67^**	Videoconference by Skype	3× weekly	To study the effects of tele-exercise on physical fitness, quality of life, loneliness and mood of older individuals in isolation during the coronavirus pandemic.	6 weeks	TUG*** STS	TUG ++: significant time effect (*P *= .017); significant group by time interaction (*P* = .01) STS ++: significant time effect (*P *= .001); significant group by time interaction (*P *≤* .001*)	N = 15, 67.1 (±3.7)	N = 15, 69.3 (±5.6)
**An et al.** **^56^**	Videoconference setup details not reported	2× working day	To investigate the effects of two separate pre-operative telerehabilitation programmes for 3 weeks in patients undergoing TKA	3 weeks	TUG	TUG ++: significant group by time interaction (*P* ≤ .001)	N = 18, 71.1 (±3.3)^1^ N = 17, 70.1 (±2.4)^2^	N = 18, 70.4 (±2.6)
**Bernocchi et al.** **^62^**	Telephone (voice) and peripherals	1× weekly	To investigate the feasibility and efficacy of a telerehabilitation programme compared to conventional care COPD and CHF patients	4 months	6MWD	6MWD ++: significant group by time interaction (*P* = .0001)	N = 56, 71 (±9)	N = 56, 70 (±9.5)
**Bernocchi et al.** **^61^**	Telephone (voice) Videoconference setup not reported	1× weekly 2× monthly	To investigate the feasibility and efficacy of a telerehabilitation programme in community-dwelling elders with fall risk and 1 or more chronic condition*	6 months	BBS TUG	BBS ++: significant group by time interaction (*P* ≤ .001) TUG ++: significant group by time interaction (*P* ≤ .001)	N = 141, 77.9 (±6.0)	N = 142, 79.3 (±7.0)
**Chaplin et al.** **^68^**	Telephone (voice) with email-based communications	1× weekly	To determine if an interactive web-based pulmonary rehabilitation programme is a feasible alternative to conventional pulmonary rehabilitation in COPD patients	11 ± 4 weeks based on milestones	ESWT ISWT	ESWT +: significant time effect (*P *≤ .01); no significant between-group difference (*P* NR) ISWT Δ: no significant time effect (*P* NR); no significant between-group difference (*P* NR)	N = 51, 66.4 (±10.1)	N = 52, 66.1 (±8.1)
**Chen et al.** **^20^**	Custom videoconferencing setup	2× working day	To determine the effectiveness of home-based telesupervising rehabilitation on function recovery for stroke patients with hemiplegia	12 weeks	BBS	BBS +: significant time effect (*P *≤ .001); no significant group by time interaction (*P *= .912)	N = 27, 66.5 (±12.1)	N = 27, 66.2 (±12.3)
**Doiron-Cadrin et al.** **^64^**	Ipad with videoconferencing software	2× weekly	To evaluate the feasibility and potential impact of a teleprehabilitation programme for patients awaiting a THA or TKA, compared to in-person prehabilitation or usual care	12 weeks	TUG	TUG Δ: no significant time effect (*P* NR); no significant group by time interaction (*P* = .282)	N = 11, 69.9 (±9.1)	N = 11, 66.7 (±9.2) N = 11, 61.3 (±8.1)**
**Gandolfi et al.** **^18^**	Videoconference by laptop. Nintendo Wii + peripherals used	3× weekly	To compare improvements in postural stability after in-home virtual reality-based balance training and in-clinic training in PD patients	7 weeks	BBS	BBS +: significant time effect (*P *≤ .001); no significant group-time effect (*P* = NR)	N = 38, 67 (±7)	N = 38, 70 (±9)
**Goode et al.** **^52^**	Telephone (voice)	1× weekly	To evaluate the effects of two home-based telephone-supported physical activity programmes for older adults with chronic lower back pain	12 weeks	TUG	TUG +: significant time effect (*P* = NR, based on Cohen *d .28-.31*) ****	N = 20, 69.6 (±3.5)^1^ N = 20, 69.5 (±4.0)^2^	N = 20, 71.9 (±6.5)
**Hansen et al.** **^69^**	Custom videoconferencing setup	3× weekly	To investigate whether pulmonary telerehabilitation is superior to conventional pulmonary rehabilitation in COPD patients	10 weeks	6MWD STS	6MWD +: significant time effect (*P* < .05); no significant group by time interaction (*P* NR) STS +: significant time effect (*P* < .05); no significant group by time interaction (*P* NR)	N = 67, 68.4 (±8.7)	N = 67, 68.2, (±9.4)
**Holland et al.** **^58^**	Telephone (voice)	1× weekly	To assess whether home-based pulmonary rehabilitation has equivalent outcomes to centre-based rehabilitation in COPD patients	8 weeks	6MWD	6MWD Δ: no significant group by time interaction (*P* NR) ****	N = 80, 69 (±13)	N = 86, 69 (±10)
**Hong et al.** **^54^**	Custom videoconferencing setup	3× weekly	To investigate the effects of tele-exercise on improvement of sarcopenia-related factors and functional fitness among community-dwelling elderly	12 weeks	TUG*** STS	TUG Δ: no significant time effect (*P *= .183); no significant group by time interaction (*P* = .956) STS +: significant time effect (*P *= .035); no significant group by time interaction (*P *= .158)	N = 11 82.2 (±5.6)	N = 12, 81.5 (±4.4)
**Hong et al.** **^55^**	Tablet with videoconferencing software	3× weekly	To evaluate the effects of a 12-week telepresence exercise programme on fall related risk factors in community-dwelling elderly women with a high risk of falling	12 weeks	BBS TUG*** STS	BBS ++: significant group by time interaction (*P* = .03) TUG Δ: no significant time effect (*P* NR); no significant group by time interaction (*P* = .40) STS ++: significant group by time interaction (*P* ≤ .001)	N = 15, 78.1 ± 5.66	N = 15, 81.54 ± 5.07
**Hwang et al.** **^59^**	Videoconference by laptop and peripherals	2× weekly	To determine if a home-based telerehabilitation programme is non-inferior to a traditional centre-based programme in CHF patients	12 weeks	6MWD TUG 10MWT	6MWD +: significant time effect (*P *= .048); no significant group by time interaction (*P *= .24) TUG Δ: no significant time effect (*P* NR); no significant group by time interaction (*P* NR) 10MWT Δ: No significant time effect (*P* NR); No significant group by time interaction (*P* NR)	N = 24, 68 (±14)	N = 29, 67 (±11)
**Li et al.** **^65^**	Unclear, guidance by ‘WeChat’ app	1× daily	To evaluate the safety and efficacy of a 6-week home-based online supervised exercise programme	6 weeks	TUG 6MWD STS	TUG +: significant time effect (*P* = .001, 95% CI 0.3–1.2). 6MWD ++: significant group by time interaction (*P* = .001) STS ++: significant group by time interaction (*P* ≤ .001)	N = 47, 65.3 (±8.7)	N = 48, 67.7 (±7.6)
**Light et al.** **^53^**	Telephone (voice)	1× weekly	To determine the effect of a home-based rehabilitation programme + telephone calls on balance control in older adults at risk of falling	12 weeks	BBS	BBS ++: significant group by time interaction (*P* = .039)	N = NR	N = NR
**Lundgren et al.** **^70^**	Videoconference by IPad	2× weekly	To determine whether home-based telerehabilitation could increase physical activity in CHF patients	12 weeks	6MWD	6MWD +: significant time effect (*P* = .02), no significant group by time interaction (*P* = .741)	N = 31, 67.6 (±10.9)	N = 30, 67.7 (±11.9)
**Menengi et al.** **^66^**	Videoconferencing by PC or tablet using Zoom	4–5× weekly	To investigate the effectiveness of exercise treatment via home-based telerehabilitation in AD patients	6 weeks	TUG	TUG: ++: significant group by time interaction (*P *= .002)	N = 10, 77.7 (±5.3)	N = 10, 80.6 (±6.1)
**Moffet et al.** **^63^**	Custom videoconferencing setup	2× weekly	To determine whether in-home telerehabilitation following a TKA, is equivalent to a face-to-face home visit approach	2 months	6MWD	6MWD Δ: no significant group by time interaction (*P* NR)****	N = 104, 65 (±8)	N = 101, 67 (±8)
**Peng et al.** **^19^**	Telephone (app) software with webcam support	3–5× weekly	To examine the effects of a telehealth exercise training programme on health outcomes in CHF patients	8 weeks	6MWD	6MWD ++: significant group by time interaction (*P* ≤ .01)	N = 49, NR Mean study age was 66.3 (±10.5)	N = 49, NR Mean study age was 66.3 (±10.5)
**Prvu Bettger et al.** **^50^**	Custom videoconferencing setup	1× weekly	To examine costs and clinical non-inferiority of a virtual physical therapy programme compared with traditional therapy after TKA	12 weeks	10MWT (after 6 weeks)	10MWT Δ: no significant time effect (*P* NR); no significant group by time interaction (*P* = .199)	N = 151, 65.4 (±7.7)	N = 153, 65.1 (±9.2)
**Tousignant et al.** **^17^**	Custom videoconferencing setup	2× weekly	To investigate the clinical efficacy of telerehabilitation at home for patients following discharge after total TKA	8 weeks	BBS TUG STS	BBS +: significant time effect (*P* NR); no significant group by time interaction (*P* NR) TUG +: significant time effect (*P* NR); no significant group by time interaction (*P* NR) STS +: significant time effect (*P* NR); no significant group by time interaction (*P* NR)	N = 21, 66 (±10)	N = 20, 66 (±13)
**Tsai et al.** **^60^**	Videoconference by laptop and peripherals	3× weekly	To examine the effectiveness of videoconferencing telerehabilitation on improving exercise capacity in patients with COPD compared with usual care	8 weeks	ESWT ISWT 6MWD	ESWT ++: significant group by time interaction (*P* ≤ .001) ISWT Δ: no significant time effect (*P* NR); no significant group by time interaction (*P* = .66) 6MWD +: significant time effect (*P* NR); no significant group by time interaction (*P* = .16)	N = 19, 73 (±8)	N = 17, 75 (±9)
**Wu et al.** **^51^**	Custom videoconferencing setup	3× weekly	To examine the effectiveness of a Tai-Chi tele-exercise programme among community-dwelling elders at risk for falls	15 weeks	TUG	TUG Δ: no significant time effect (*P* = .60); no significant between-group difference (*P* = .80)	N = 22, 76.1 (±7.9)	N = 20, 74.1 (±6.9) N = 22, 75.9 (±6.3)**
**Yerlikaya et al.** **^71^**	Videoconference. Setup details not reported	3× weekly	To investigate the effectiveness of a home-based interactive telerehabilitation programme with a non-supervised home exercise programme in an older population	8 weeks	BBS TUG	BBS +: significant time effect (*P *≤ .001) TUG +: significant time effect (*P *≤ .001)	N = 18, 70.2 (±5.5)	N = 16, 75.6 (±8.7) N = 16, 71.8 (±6.6)**
**Yi and Yim** **^57^**	Live streaming by smartphone with a-synchronous telephone contact	2× weekly	To compare the effects of a remote home-based exercise programme to improve the mental state, balance, physical function and to prevent falls in adults aged 65 years and older	8 weeks	TUG 10MWT	TUG ++: significant group by time interaction (*P* = .009) 10MWT ++: significant group by time interaction (*P* = .003)	N = 35, 76.1 (±6.3)	N = 35, 77.3 (±5.6)

Main findings from reported results: ++ = Telerehabilitation has a significant group by time interaction, indicating superiority over the control intervention. + = Telerehabilitation only has a significant time effect, indicating non-superiority over the control intervention. Δ = Telerehabilitation has a non-significant time effect and a non-significant group by time interaction (or a non-significant between group difference if no interaction reported), indicating non-inferiority with the control intervention.

*P*-value is significant if < .05;^1 ^= Study intervention group 1; ^2 ^= Study intervention group 2.

‡ Findings are summarised to main effects by statistical significance. Exact effect measures are reported in the Supplemental Table 3.

*Chronic cardiac, respiratory, neurological or neuromuscular condition(s). **Respective study intervention group, but considered control as it did not meet criteria of this review. ***Eight-foot up and go test was equated with the TUG. ****Results determined in respective study by mean difference with 95% confidence intervals.

6MWD: 6-minute Walking Distance test; 10MWT: 10 m walk test; AD: Alzheimer's disease; BBS: Berg Balance Scale; CHF: chronic heart failure; COPD: chronic obstructive pulmonary disorder; DM: diabetes mellitus; ESWT: Endurance Shuttle Walk test; FP: functional performance; ISWT: Incremental Shuttle Walk test; N: number of participants; NR: not reported; OA: osteoarthritis; PD: Parkinson's disease; SD: standard deviation; STS:30 s sit to stand test; THA: total hip arthroplasty; TKA: total knee arthroplasty; TUG: Timed-Up-and-Go test.

Between-study differences in intervention characteristics, such as pre-intervention training, intervention and study duration, intervention frequency, guidance frequency, guidance synchronicity, intervention contents and intensity, individual-based or group-based intervention, etc., contributed greatly to significant clinical heterogeneity. This significant clinical heterogeneity precluded meta-analysis. We therefore qualitatively evaluated the study's primary outcomes and the estimate of effect by providing a comprehensive narrative summary. The following summary of intervention characteristics of included studies is presented.

Telerehabilitation guidance was either synchronously or a-synchronously with the study intervention. Sixteen studies applied synchronous two-way videoconferencing as the mode of delivery for telerehabilitation,^17,18,20,50,51,54,55,59,60,63,64,66,67,69–71^ while six studies used a-synchronous modes of delivery, namely telephone (voice) communication,^52,53,58,62^ telephone (app)^65^ and telephone (voice) with email-based communication.^68^ Three studies applied a combination of synchronous and a-synchronous guidance methods, namely telephone (voice) communication supported by videoconference,^57,61^ and telephone (app) communication with two-way webcam support.^19^ Lastly, one study used two intervention groups, one with synchronous (videoconferencing) and one with an a-synchronous (telephone (voice)) guidance method.^56^ Two studies applied additional modalities, namely videoconferencing combined with a Nintendo-Wii console^18^ and videoconferencing combined with virtual reality.^50^

Telerehabilitation guidance frequency ranged from as often as two times daily to the minimum of once weekly. Nine studies had a guidance frequency of three times weekly,^18,19,51,54,55,60,67,69,71^ six studies had a guidance frequency of two times weekly,^17,57,59,63,64,70^ seven studies had a guidance frequency of once weekly^50,52,53,58,61,62,68^ and two studies had a guidance frequency of two times daily.^20,56^ Study intervention durations varied from 3 weeks to 6 months, with the majority being 12 weeks.^20,50,52–55,59,64,70^ The duration of individual rehabilitation sessions varied from 20 to 60 minutes, with the majority being 60 minutes.^17,20,51,59,60,63,69,70^

Rehabilitation contents varied across studies: a combination of endurance and resistance training,^19,58–62,65,68,70^ a combination of exercises for improving function,^17,56,57,63,64^ a combination of resistance, endurance and balance training,^53^ a combination of resistance, balance training and stretching,^67^ a combination of resistance and balance training,^55,71^ a combination of resistance training and stretching,^52^ only resistance training,^54^ only endurance training,^69^ only balance training,^18^ a combination of chair-based exercises with cognitive tasks,^66^ a combination of physical exercise with electromyography-triggered neuromuscular stimulation technique,^20^ Tai-Chi specific exercises^51^ and one study was unclear about the specifics of exercise components.^50^ Lastly, one study specifically mentioned using cognitive behavioural techniques in the rehabilitation intervention.^52^

Ten studies^17–20,53,59,61,62,66,70^ accounted for safety measures during telerehabilitation interventions, which generally required a family member or caregiver to be present to assist if necessary. Lundgren et al.^70^ described performing pre-intervention exercise testing and monitoring all exercises performed real-time in front of the tablet computer, while having addresses and telephone numbers readily available. Bernocchi et al.^61^ also had a nurse tutor available 24h a day in the case of urgent need or an emergency. In two studies,^19,60^ a heart rate monitor or an SpO_2_ device was provided in order to monitor participants from a distance.

Ten studies^18,20,50,58–61,68,70,71^ provided pre-intervention training or familiarity sessions with the intervention technology. Hong et al.^54,55^ described providing a PC-operation manual and TeamViewer remote assistance but no in-person information session, while Tousignant et al.^17^ provided family member training for technology familiarity.

Fifteen studies used usual care for their studies’ respective medical condition as the control group type.^17,20,50,51,53,56,58,59,61–64,67–70^ One study applied in-clinic sensory integration training as the control group,^18^ one study applied general advice with regular clinical follow-ups,^19^ one study provided in-person health education and a non-supervised home exercise programme,^65^ one study provided usual medical management without exercise training,^60^ while seven studies used daily routines without any interventions as the control group.^52,54,55,57,66,67,71^

Telerehabilitation interventions were group based in eight studies,^18,51,57,59,60,69–71^ with all other studies having individual guidance contact. Four studies^50,61,63,64^ specified to combine telerehabilitation with face-to-face therapy in case of particular needs, the remaining studies did not or made no mention of providing it.

Adverse events were reported in 17 studies,^18–20,50,52,58–67,69,70^ of which 11 studies^18–20,52,58,61,62,65–67,70^ described no adverse events occurred during the telerehabilitation intervention. The adverse events in the remaining six studies^50,59,60,63,64,69^ were described as minor or unrelated to the telerehabilitation intervention and were not significantly different between intervention and control groups.

### Effectiveness of telerehabilitation

Effectiveness of functional performance outcome measures for the telerehabilitation intervention compared to the control intervention are presented in Table 1. Findings are summarised to main effects by statistical significance. Full exact effect measures are presented in the Supplemental files (Table 3).

Results on effectiveness are presented on each reported outcome measure separately (n = 43). Eleven studies (16 outcome measures) found a significant group by time interaction for the telerehabilitation intervention, indicating superiority over the control intervention.^19,53,55–57,60–62,65–67^ Twelve studies (16 outcome measures) found a significant improvement over time for the telerehabilitation intervention, with no (reported) significant group by time interaction, indicating non-superiority over the control intervention.^17,18,20,52,54,59,60,65,68–71^ Nine studies (11 outcome measures) found no significant improvement over time for the telerehabilitation intervention, with no (reported) significant group by time interaction, indicating non-inferiority with the control intervention.^50,51,54,55,58,60,63,64,68^ Lastly, no studies found a significant group by time interaction in favour of the control intervention, indicating no studies found the control intervention to be superior over the telerehabilitation intervention.

### Methodological quality

**Figure 2.**
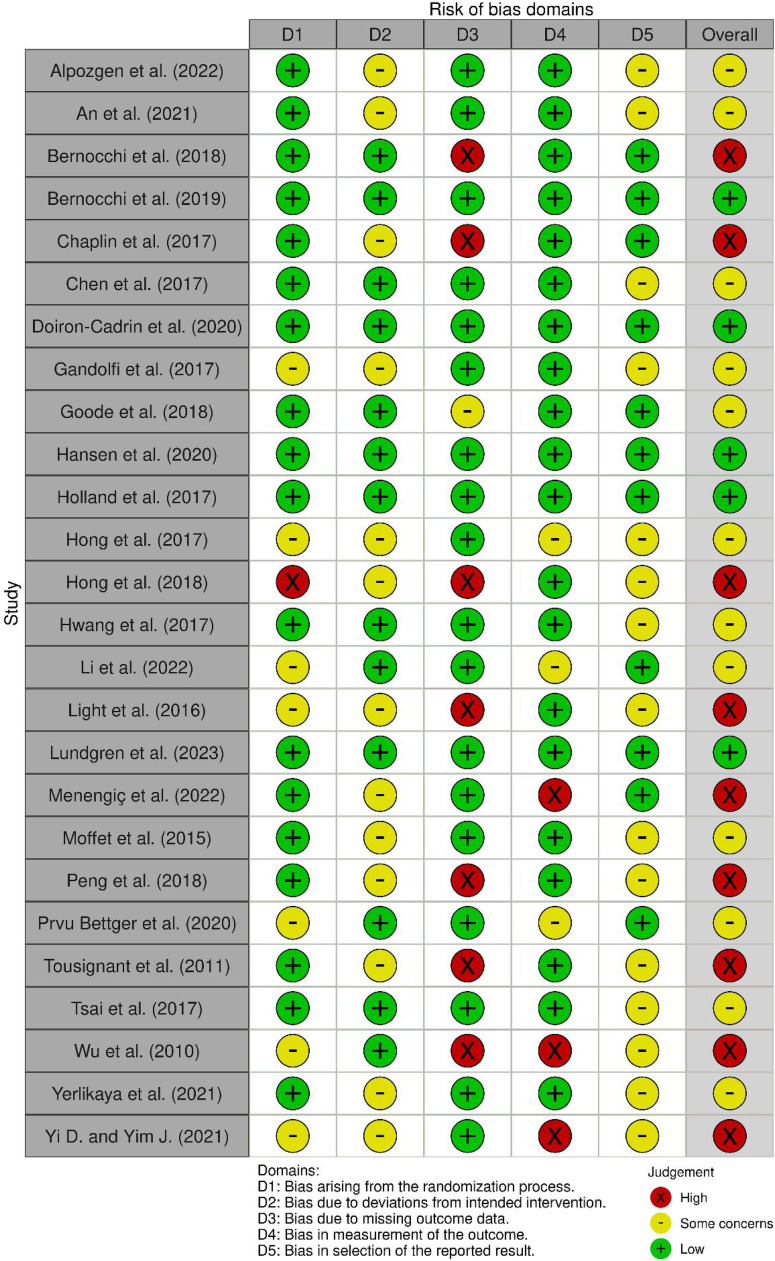
Summary of the revised Cochrane Risk-of-Bias 2 tool for randomised trials.^49^.

Figure 2 summarises the results of the risk of bias assessment using the revised Cochrane Risk-of-Bias tool for randomised trials.^49^ Five studies were found to present an overall ‘low’ risk of bias, 12 studies to present ‘some’ risk of bias and 9 studies to present an overall ‘high’ risk of bias. The quality of evidence was low-to-moderate across each of the three tiers of effectiveness in included studies. The most common methodological limitations were in domains 2 and 5. Due to the nature of the intervention, blinding of participants and healthcare professionals was mostly not possible, which is a known challenge for telerehabilitation trials.^72^ Therefore, no points on methodological quality were deducted due to this reason. More information on the risk of bias assessment is found in the Supplemental files (Table 4).

## Discussion

Twenty-six randomised controlled trials reporting on the effectiveness of guided telerehabilitation for improving functional performance in community-dwelling older adults were identified in this review. Eleven studies found the effect measures of telerehabilitation to be significantly superior to the control intervention, while no studies found the control intervention to be superior over telerehabilitation. These findings suggest that telerehabilitation can be effective over time for improving functional performance in community-dwelling older adults and could be a capable alternative to conventional in-person care. However, the methodological quality of evidence was low-to-moderate, indicating caution when interpreting these findings.

This review adds to the findings of previous reviews evaluating the effectiveness of telerehabilitation in community-dwelling older adults.^7,32–35^ Our findings are in accordance with Batsis et al.^34^ and Saito et al.,^32^ who found telerehabilitation capable of reaching similar or superior outcomes compared to conventional rehabilitation interventions, yet emphasised the need for well-designed studies with minimised bias. However, this review is the first to use a broad definition of telerehabilitation, to adhere to current clinical guidelines and use no inclusion criteria for geographical barriers.

Currently, no evidence-based guideline exists for providing telerehabilitation interventions to community-dwelling older adults. Knowledge is lacking of which intervention characteristics constitute effective telerehabilitation intervention strategies.^12^ This has become apparent by the vast array of intervention characteristics throughout included studies. When considering the characteristic of guidance, for example, we have specifically chosen to include those studies that follow current (in-person) clinical guidelines on guidance frequency criteria for their respective medical condition. Reviews by Batsis et al.^34^ and Cottrell et al.^27^ have attempted to specify another key intervention characteristic, namely guidance synchronicity. Batsis et al.^34^ defined ‘ambulatory telemedicine’ as live, synchronous two-way videoconferencing under the premise this was most commonly used and reimbursed. Cottrell et al.^27^ only included trials utilising synchronous telerehabilitation mediums, explaining this method is more reflective of conventional face-to-face interventions. For several trials within the current review,^17,20,51,59,63^ the synchronous guided exercise protocols were identical between both the telerehabilitation and control groups. Thus, any difference in results should be considered as a reflection of the method of treatment delivery. Additionally, considering in-person interventions are currently still favoured by the older adult population,^73^ it might explain why synchronous guided telerehabilitation interventions are found in 71% of studies included in the current review. In contrast, Bini et al.^74^ argues that attempts to recreate in-person interventions digitally will inevitably fall short, due to methodological, technical and planning issues. Instead, independently performed interventions with a-synchronous guidance are likely easier to implement, less costly and more effective than synchronous guidance strategies.^74^ Moreover, a-synchronous guided interventions provide the most flexibility for rehabilitation at ‘any place at any time’. Still, the impact of specific methods of guidance synchronicity on the effectiveness of telerehabilitation interventions has not been determined.^75^ Similarly, the current review found heterogeneous intervention characteristics across all levels of methodological quality evidence and effect measure sizes. Accordingly, the impact of these characteristics on the effectiveness of telerehabilitation could not be determined.

We acknowledge several strengths and limitations for the evidence identified within this review. By using the PRISMA criteria, we reduced inherent bias and error that are present in conducting systematic reviews. Moreover, by utilising the revised Cochrane Risk-of-Bias tool for randomised trials, deficiencies in methodological quality and standards of reporting were revealed. However, this review also has some limitations. First, publication and selection bias might be present. Since the search strategy was performed in three databases, it is possible that some publications may have been missed. Selection bias in included studies could be present as all but four studies excluded participants with exercise limiting conditions due to comorbidities, other than the primary condition. Moreover, three studies had specific digital literacy inclusion criteria for participants, which might impede the generalisability of results for the community-dwelling older adult population. Second, studies were selected using a specified set of outcome measures to cover the construct of functional performance. As this set was not all encompassing, it is possible that some publications were omitted. Third, significant clinical heterogeneity, particularly regarding the abundance of intervention characteristics, precluded meta-analysis, which might have strengthened the results. Lastly, the risk of bias assessment scored ‘*high*’ or ‘*some concerns*’ for bias in 21 included studies (81%). However, five of these studies were only missing data in one domain, with the remaining domains scoring ‘*no risk*’ of bias. This may have caused the overall risk of bias score to be skewed towards unfavourable, potentially diminishing the impact of bias in included studies.

While the findings of this study underscore the potential of telerehabilitation, more well-designed studies are needed to provide definitive findings on the effectiveness of telerehabilitation interventions for improving functional performance in community-dwelling older adults. Further understanding on the impact of potentially relevant intervention characteristics is needed to add clarity to future studies on telerehabilitation and help form guidelines for healthcare practise.^76^ Moreover, no studies included in this review applied routine in-person visits for the intervention groups. With the increase in frail community-dwelling older adults, the future of geriatric telerehabilitation will likely involve a balance of in-person and remote care.^12^ Lastly, the contents and results of this review are presented at a tipping point in time following the impact of the COVID-19 pandemic. Rules, regulations and policies were altered to enable widespread use of technology-supported healthcare interventions as opposed to in-person visits.^77,78^ These circumstances offer key opportunities to further investigate whether telerehabilitation has the potential of becoming a sustainable solution for the aging population with need for rehabilitation services.

**Clinical messages**

- As the global population age increases, integrating technology-driven interventions like telerehabilitation in elderly care can increase accessibility and ultimately contribute to improved outcomes for older adults with need for rehabilitation services.
- Uniformly conceptualised intervention characteristics can add clarity to future studies on telerehabilitation and help form guidelines for healthcare practise.
- Well-designed telerehabilitation research is lacking in community-dwelling older adults, particularly when combined with in-person visits.
- Collaborative efforts between the fields of research, clinicians and technology developers are critical to optimise the telerehabilitation landscape for older adults.

## Supplemental Material

sj-docx-1-cre-10.1177_02692155231217411 - Supplemental material for Effectiveness of guided telerehabilitation on functional performance in community-dwelling older adults: A systematic review Supplemental material, sj-docx-1-cre-10.1177_02692155231217411 for Effectiveness of guided telerehabilitation on functional performance in community-dwelling older adults: A systematic review by CJ Gamble, JCM van Haastregt, EF van Dam van Isselt, SMG Zwakhalen and JMGA Schols in Clinical Rehabilitation
