# Supplementary material for: Effectiveness of guided telerehabilitation on functional performance in community-dwelling older adults: A systematic review
Source: Clin Rehabil. 2023 Nov 28;38(4):457–77. doi: 10.1177/02692155231217411 (PMC10898211; doi:10.1177/02692155231217411)
Supplement: sj-docx-1-cre-10.1177_02692155231217411 - Supplemental material for Effectiveness of guided telerehabilitation on functional performance in community-dwelling older adults: A systematic review [file sj-docx-1-cre-10.1177_02692155231217411.docx]

**Supplementary files**

**page #**

**Search strategy Medline…………………………………………………………………………………………………………………….2

Search strategy CENTRAL……………………………………………………………………………………………………………………3

Search strategy Embase……………………………………………………………………………………………………………………..4

Prisma Checklist..……………………………………………………………………………………………………………………………….5

Additional study information**

**Table 1……………………………………………………………………………………………………………………………………8**

**Table 2..…………………………………………………………………………………………………………………………………11**

**Table 3..…………………………………………………………………………………………………………………………………14**

**Revised Cochrane Risk-Of-Bias tool assessment**

**Table 4…………….…………………………………………………………………………………………………………………….18**

**Search strategy Medline:**

(telerehabilitation[MeSH] OR telerehabilitation[tw] OR tele-rehabilitation[tw] OR e-rehabilitation[tw] OR telerehab[tw] OR tele-rehab[tw] OR tele-exercise[tw] OR telegeriatrics[tw])

**OR**

((remote[tiab] OR online[tiab] OR web[tiab] OR video[tiab] OR virtual[tiab] OR mobile[tiab] OR phone[tiab] OR internet[tiab] OR technology[tiab] OR technologies[tiab] OR telemedicine[MeSH] OR telemedicine[tiab] OR telehealth[tiab] OR mhealth[tiab] OR e-health[tiab] OR ehealth[tiab] OR videoconferencing[MeSH] OR videoconferencing[tiab]) **AND** (rehabilitation[MeSH] OR rehabilitation[tiab] OR aftercare[MeSH] OR “after care”[tiab] OR after-treatment[tiab] OR “after treatment”[tiab] OR after-treatments[tiab] OR “follow-up care”[tiab] OR “follow up care”[tiab] OR “follow-up cares”[tiab] OR “exercise therapy”[MeSH] OR remedial exercis*[tiab] OR “exercise therapies”[tiab] OR rehabilitation exercis*[tiab] OR “postoperative care”[MeSH] OR “postoperative care”[tiab]))

**AND**

(aged[MeSH] OR "aged, 80 and over"[MeSH] OR “frail elderly”[MeSH] OR "health services for the aged"[Mesh Terms] OR geriatrics[MeSH] OR elderly[tw] OR senior[tw] OR geriatric[tw] OR frail[tw] OR old age*[tw] OR septuagenarian*[tw] OR octagenarian*[tw] OR octogenarian*[tw] OR nonagenarian*[tw] OR centarian*[tw] OR centenarian*[tw] OR supercentenarian*[tw] OR older people[tw] OR older subject*[tw] OR older patient*[tw] OR older age*[tw] OR older adult*[tw] OR older man[tw] OR older men[tw] OR older male*[tw] OR older woman[tw] OR older women[tw] OR older female*[tw] OR older population*[tw] OR older person*[tw] OR "ages 65"[tiab] OR "aged 65"[tiab] OR "65 and over"[tiab])

**AND**

("2010/01/01"[PDAT] : "2020/12/31"[PDAT])

**AND**

(randomized controlled trial[pt] OR "randomized controlled trials as topic"[MeSH] OR "random allocation"[MeSH] OR "double-blind method"[MeSH] OR "single-blind method"[MeSH] OR randomized[tiab] OR placebo[tiab] OR randomly[tiab] OR random[tiab] OR trial[tiab] OR groups[tiab] NOT ("animals"[MeSH] NOT "humans"[MeSH]))

* End date edited to ‘current’ when updating searches.

**Search strategy CENTRAL**

#1 MeSH descriptor: [Telerehabilitation] explode all trees

#2 (telerehabilitation OR tele-rehabilitation OR e-rehabilitation OR telerehab OR tele-rehab OR tele-exercise OR telegeriatrics) (Word variations have been searched)

#3 #1 OR #2

#4 (remote OR online OR web OR video OR virtual OR mobile OR phone OR internet OR technology OR telemedicine OR telehealth OR mhealth OR e-health OR ehealth OR videoconferencing):ti,ab,kw (Word variations have been searched)

#5 MeSH descriptor: [Telecommunications] explode all trees

#6 MeSH descriptor: [Telemedicine] explode all trees

#7 MeSH descriptor: [Remote Consultation] explode all trees

#8 MeSH descriptor: [Aftercare] explode all trees

#9 MeSH descriptor: [Rehabilitation] explode all trees

#10 MeSH descriptor: [Postoperative Care] explode all trees

#11 (“after care” OR after-treatment OR “follow-up care” OR “exercise therapy” OR remedial exercise OR rehabilitation exercise OR “postoperative care”):ti,ab,kw (Word variations have been searched)

#12 (#4 OR #5 OR #6 OR #7) AND (#8 OR #9 OR #10 OR #11)

#13 MeSH descriptor: [Aged] explode all trees

#14 MeSH descriptor: [Aged, 80 and over] explode all trees

#15 MeSH descriptor: [Frail Elderly] explode all trees

#16 MeSH descriptor: [Health Services for the Aged] explode all trees

#17 MeSH descriptor: [Geriatrics] explode all trees

#18 (elderly OR senior OR geriatric OR frail OR "old age" OR septuagenarian OR octagenarian OR octogenarian OR nonagenarian OR centarian OR centenarian OR supercentenarian OR "older people" OR "older subject" OR "older patient" OR "older age" OR "older adult" OR "older man" OR "older woman" OR "older population" OR "older person" OR "ages 65" OR "aged 65" OR "65 and over") (Word variations have been searched)

#19 #13 OR #14 OR #15 OR #16 OR #17 OR #18

#20 (#3 OR #12) AND #19 with Publication Year from 2010 to 2020, with Cochrane Library publication date Between Jan 2010 and Dec 2020, in Trials

* End date edited to ‘current’ when updating searches.

**Search strategy Embase**

| 1. exp telerehabilitation/ |  | |
| --- | --- | --- |
| 2. (telerehabilitation or telerehab or tele-rehab or telegeriatrics or tele-geriatrics).mp. |  | |
| 3. 1 or 2 |  | |
| 4. (remote or online or web or video or virtual or mobile or phone or internet or technology or technologies or digital or telemedicine or telehealth or mhealth or e-health or ehealth or videoconferencing).ti,ab. |  | |
| 5. exp telemedicine/ or exp telehealth/ or exp teletherapy/ or telecommunication/ or exp videoconferencing/ or exp teleconsultation/ or exp teleconference/ or exp wireless communication/ |  | |
| 6. exp rehabilitation/ or exp aftercare/ or exp postoperative care/ or exp kinesiotherapy/ |  | |
| 7. (rehabilitation or ((after or postoperative or follow-up) adj1 care) or ((exercis* or fitness or physical or functional) adj2 (therap* or training or treatment))).ti,ab. |  | |
| 8. (4 or 5) and (6 or 7) |  | |
| 9. exp aged/ or geriatrics/ |  | |
| 10. (elderly or senior or geriatric or septuagenarian* or octagenarian* or octogenarian* or nonagenarian* or centarian* or centenarian* or supercentenarian* or (older and (people or subject* or patient* or age or adult* or m#n or wom#n or population)) or ((ages or aged) adj2 "65")).ti,ab. |  | |
| 11. 9 or 10 |  | |
| 12. (3 or 8) and 11 |  | |
| 13. Randomized controlled trial/ or ((random$.ti,ab. or randomization/ or intermethod comparison/ or ((double or single or doubly or singly) adj (blind or blinded or blindly)).ti,ab. or double blind procedure/ or parallel group$1.ti,ab. or ((assign$ or match or matched or allocation) adj5 (alternate or group$1 or intervention$1 or patient$1 or subject$1 or participant$1)).ti,ab. or (controlled adj7 (study or design or trial)).ti,ab. or human experiment/ or trial.ti.) not (((random$ adj sampl$ adj7 ("cross section$" or questionnaire$1 or survey$ or database$1)).ti,ab. not (comparative study/ or cohort study/ or controlled study/ or randomi?ed controlled.ti,ab. or randomly assigned.ti,ab.)) or (Cross-sectional study/ not (randomized controlled trial/ or controlled study/ or randomi?ed controlled.ti,ab. or control group$1.ti,ab.)) or (((case adj control$) and random$) not randomi?ed controlled).ti,ab. or (Systematic review not (trial or study)).ti. or (nonrandom$ not random$).ti,ab. or "Random field$".ti,ab. or (random cluster adj3 sampl$).ti,ab. or ((review.ab. and review.pt.) not trial.ti.) or ("we searched".ab. and (review.ti. or review.pt.)) or "update review".ab. or (databases adj4 searched).ab. or ((rat or rats or mouse or mice or swine or porcine or murine or sheep or lambs or pigs or piglets or rabbit or rabbits or cat or cats or dog or dogs or cattle or bovine or monkey or monkeys or trout or marmoset$1).ti. and animal experiment/) or (Animal experiment/ not (human experiment/ or human/)))) |  | |
| 14. 12 and 13 |  | |
| 15. limit 14 to yr="2010 -Current"  * End date edited to ‘current’ when updating searches. |  |  |

**PRISMA Checklist**

| **Section and Topic** | **Item #** | **Checklist item** | **Location where item is reported** |
| --- | --- | --- | --- |
| **TITLE** | | |  |
| Title | 1 | Identify the report as a systematic review. | **1** |
| **ABSTRACT** | | |  |
| Abstract | 2 | See the PRISMA 2020 for Abstracts checklist. | **2** |
| **INTRODUCTION** | | |  |
| Rationale | 3 | Describe the rationale for the review in the context of existing knowledge. | **3,4** |
| Objectives | 4 | Provide an explicit statement of the objective(s) or question(s) the review addresses. | **4** |
| **METHODS** | | |  |
| Eligibility criteria | 5 | Specify the inclusion and exclusion criteria for the review and how studies were grouped for the syntheses. | **5-7** |
| Information sources | 6 | Specify all databases, registers, websites, organisations, reference lists and other sources searched or consulted to identify studies. Specify the date when each source was last searched or consulted. | **5** |
| Search strategy | 7 | Present the full search strategies for all databases, registers and websites, including any filters and limits used. | **5 + supplementary files** |
| Selection process | 8 | Specify the methods used to decide whether a study met the inclusion criteria of the review, including how many reviewers screened each record and each report retrieved, whether they worked independently, and if applicable, details of automation tools used in the process. | **6,7** |
| Data collection process | 9 | Specify the methods used to collect data from reports, including how many reviewers collected data from each report, whether they worked independently, any processes for obtaining or confirming data from study investigators, and if applicable, details of automation tools used in the process. | **6,7** |
| Data items | 10a | List and define all outcomes for which data were sought. Specify whether all results that were compatible with each outcome domain in each study were sought (e.g. for all measures, time points, analyses), and if not, the methods used to decide which results to collect. | **6,7** |
|  | 10b | List and define all other variables for which data were sought (e.g. participant and intervention characteristics, funding sources). Describe any assumptions made about any missing or unclear information. | **6** |
| Study risk of bias assessment | 11 | Specify the methods used to assess risk of bias in the included studies, including details of the tool(s) used, how many reviewers assessed each study and whether they worked independently, and if applicable, details of automation tools used in the process. | **8** |
| Effect measures | 12 | Specify for each outcome the effect measure(s) (e.g. risk ratio, mean difference) used in the synthesis or presentation of results. | **7** |
| Synthesis methods | 13a | Describe the processes used to decide which studies were eligible for each synthesis (e.g. tabulating the study intervention characteristics and comparing against the planned groups for each synthesis (item #5)). | **7** |
|  | 13b | Describe any methods required to prepare the data for presentation or synthesis, such as handling of missing summary statistics, or data conversions. | **7** |
|  | 13c | Describe any methods used to tabulate or visually display results of individual studies and syntheses. | **7** |
|  | 13d | Describe any methods used to synthesize results and provide a rationale for the choice(s). If meta-analysis was performed, describe the model(s), method(s) to identify the presence and extent of statistical heterogeneity, and software package(s) used. | **6,7** |
|  | 13e | Describe any methods used to explore possible causes of heterogeneity among study results (e.g. subgroup analysis, meta-regression). | **7** |
|  | 13f | Describe any sensitivity analyses conducted to assess robustness of the synthesized results. | **-** |
| Reporting bias assessment | 14 | Describe any methods used to assess risk of bias due to missing results in a synthesis (arising from reporting biases). | **8** |
| Certainty assessment | 15 | Describe any methods used to assess certainty (or confidence) in the body of evidence for an outcome. | **-** |
| **RESULTS** | | |  |
| Study selection | 16a | Describe the results of the search and selection process, from the number of records identified in the search to the number of studies included in the review, ideally using a flow diagram. | **9, figure 1** |
|  | 16b | Cite studies that might appear to meet the inclusion criteria, but which were excluded, and explain why they were excluded. | **9, figure 1** |
| Study characteristics | 17 | Cite each included study and present its characteristics. | **10, table 1** |
| Risk of bias in studies | 18 | Present assessments of risk of bias for each included study. | **17,18, figure 3** |
| Results of individual studies | 19 | For all outcomes, present, for each study: (a) summary statistics for each group (where appropriate) and (b) an effect estimate and its precision (e.g. confidence/credible interval), ideally using structured tables or plots. | **Table 1 + supplementary files** |
| Results of syntheses | 20a | For each synthesis, briefly summarise the characteristics and risk of bias among contributing studies. | **-** |
|  | 20b | Present results of all statistical syntheses conducted. If meta-analysis was done, present for each the summary estimate and its precision (e.g. confidence/credible interval) and measures of statistical heterogeneity. If comparing groups, describe the direction of the effect. | **-** |
|  | 20c | Present results of all investigations of possible causes of heterogeneity among study results. | **10-12** |
|  | 20d | Present results of all sensitivity analyses conducted to assess the robustness of the synthesized results. | **-** |
| Reporting biases | 21 | Present assessments of risk of bias due to missing results (arising from reporting biases) for each synthesis assessed. | **-** |
| Certainty of evidence | 22 | Present assessments of certainty (or confidence) in the body of evidence for each outcome assessed. | **-** |
| **DISCUSSION** | | |  |
| Discussion | 23a | Provide a general interpretation of the results in the context of other evidence. | **17-22** |
|  | 23b | Discuss any limitations of the evidence included in the review. | **20,21** |
|  | 23c | Discuss any limitations of the review processes used. | **20,21** |
|  | 23d | Discuss implications of the results for practice, policy, and future research. | **21,22** |
| **OTHER INFORMATION** | | |  |
| Registration and protocol | 24a | Provide registration information for the review, including register name and registration number, or state that the review was not registered. | **5** |
|  | 24b | Indicate where the review protocol can be accessed, or state that a protocol was not prepared. | **5** |
|  | 24c | Describe and explain any amendments to information provided at registration or in the protocol. | **5** |
| Support | 25 | Describe sources of financial or non-financial support for the review, and the role of the funders or sponsors in the review. | **22** |
| Competing interests | 26 | Declare any competing interests of review authors. | **23** |
| Availability of data, code and other materials | 27 | Report which of the following are publicly available and where they can be found: template data collection forms; data extracted from included studies; data used for all analyses; analytic code; any other materials used in the review. | **Supplementary files** |

*From:*  Page MJ, McKenzie JE, Bossuyt PM, Boutron I, Hoffmann TC, Mulrow CD, et al. The PRISMA 2020 statement: an updated guideline for reporting systematic reviews. BMJ 2021;372:n71. doi: 10.1136/bmj.n71

For more information, visit: <http://www.prisma-statement.org/>

**Additional study information**

| Table 1. Study Characteristics of included Randomised Controlled Studies (n=26) | | | | | | | |
| --- | --- | --- | --- | --- | --- | --- | --- |
|  | **Telerehabilitation intervention model** | | |  |  | **Population**  **N, mean age ± SD** | |
| Study | **Mode of delivery** | **Guidance by** | **Guidance frequency and mode** | **Study aim** | **Study duration** | **Intervention** | **Control** |
| Alpozgen et al., 2022 | Videoconference by Skype. Setup details not reported. | Physiotherapist | Videoconference  45 min/session, 3x week | To study the effects of tele-exercise on physical fitness, quality of life, loneliness and mood of older individuals during the coronavirus pandemic. | 6 weeks | N= 15, 67.1 (±3.7) | N= 15, 69.3 (±5.6) |
| An et al., 2021 | Smartphone or tablet. Setup details not reported. | Physiotherapist | Videoconference  30 min/session, 2 times/day, 5 days/week. | To investigate the effects of a preoperative telerehabilitation programme for 3 weeks in patients undergoing TKA. | 3 weeks | N= 18, 71.1 (±3.3)^1^  N= 17, 70.1 (±2.4)^2^ | N= 18, 70.4 (±2.6) |
| Bernocchi et al., 2018 | Telephone (voice) and peripherals | Physiotherapist and Nurse | 1x 45min weekly by telephone | To investigate the feasibility and efficacy of a telerehabilitation programme compared to conventional care. | 4 months | N= 56, 71 (±9) | N= 56, 70 (±9.5) |
| Bernocchi et al., 2019 | Telephone (voice). Videoconference setup not reported | Physiotherapist and Nurse | 1x weekly by telephone, 2x monthly by videoconference  Average duration not reported | To investigate the feasibility and efficacy of a telerehabilitation programme compared to conventional care. | 6 months | N= 141, 77.9 (±6.0) | N= 142, 79.3 (±7.0) |
| Chaplin et al., 2017 | Website-based | Rehabilitation specialist | 1x weekly by telephone and/or email, average duration not reported | To determine if an interactive web-based pulmonary rehabilitation (PR) programme is a feasible alternative to conventional PR. | 11±4 weeks based on milestones | N= 51, 66.4 (±10.1) | N= 52, 66.1 (±8.1) |
| Chen et al., 2017 | Custom videoconferencing setup | Physiotherapist | Videoconference  2x working day 1 hour exercise.  2x working day ETNS 20min | To determine the effectiveness of home-based tele-supervising rehabilitation on function recovery for stroke patients with hemiplegia. | 12 weeks | N= 27, 66.52 (±12.08) | N= 27, 66.15 (±12.33) |
| Doiron-Cadrin et al., 2020 | Ipad with custom app or videoconferencing software e.g. Skype or FaceTime. | Physiotherapist | 2x weekly by videoconference. Average duration not reported | To evaluate the feasibility and potential impact of a tele-prehabilitation programme for patients awaiting a THA or TKA, compared to in-person prehabilitation or usual care. | 12 weeks | N=11, 69.9 (±9.1) | N=11, 66.7 (±9.2)  N=12, 61.3 (±8.1) |
| Gandolfi et al., 2017 | Laptop with built-in webcam and peripherals. Nintendo Wii console, Wii Fit en Balance Board | Physiotherapist | 3x 50min weekly sessions by videoconference | To compare improvements in postural stability after in-home VR-based balance training and in-clinic training in Parkinson’s Disease patients. | 7 weeks | N= 38, 67 (±7) | N= 38, 70 (±9) |
| Goode et al., 2018 | Telephone (voice) | Physiotherapist and exercise counselor | 1x 15min weekly by telephone | To evaluate the effects of a home-based telephone-supported physical activity programme for older adults with chronic lower back pain. | 12 weeks | N=20, 69.6 (±3.5)^1^  N=20, 69.5 (±4.0)^2^ | N=20, 71.9 (±6.5) |
| Hansen et al., 2020 | Custom videoconferencing setup | Physiotherapist and respiratory nurses | 3x 1 hour weekly sessions by videoconference | To investigate whether pulmonary tele-rehabilitation is superior to conventional pulmonary rehabilitation in patients with COPD. | 10 weeks | N=67, 68.4 (±8.7) | N=67, 68.2, (±9.4) |
| Holland et al., 2017 | Telephone (voice) | Physiotherapist | 1x weekly by telephone. Average duration not reported | To assess whether home-based pulmonary rehabilitation has equivalent outcomes to centre-based pulmonary rehabilitation. | 8 weeks | N= 80, 69 (±13) | N=86, 69 (±10) |
| Hong et al., 2017 | Custom videoconferencing setup | Exercise instructor | 3x 20-40min weekly sessions by videoconference | To investigate the effects of tele-exercise on improvement of sarcopenia-related factors and functional fitness among community-dwelling elderly. | 12 weeks | N=11 82.2 (±5.6) | N=12, 81.5 (±4.4) |
| Hong et al., 2018 | Tablet with videoconferencing software | Exercise instructor | 3x 20-40min weekly sessions by videoconference | To evaluate the effects of a 12-week telepresence exercise programme on fall related risk factors in community-dwelling elderly women with a high risk of falling. | 12 weeks | N=15, 78.1 ± 5.66 | N=15, 81.54 ± 5.07 |
| Hwang et al., 2017 | Laptop with built-in webcam and peripherals | Physiotherapist | 2x 1 hour weekly sessions by videoconference | To determine if a 12-week, home-based telerehabilitation programme is non-inferior to a traditional centre-based program. | 12 weeks | N= 24, 68 (±14) | N= 29, 67 (±11) |
| Li et al., 2022 | Unclear, contact by app WeChat | Physiotherapist | 1x daily | To evaluate the safety and efficacy of a 6-week home-based online supervised exercise programme. | 6 weeks | N= 47, 65.3 (±8.7) | N= 48, 67.7 (±7.6) |
| Light et al., 2016 | Telephone (voice) | Physiotherapist and support staff | 1x 15min weekly by telephone | To determine the effect of telephone calls on balance in older adults at risk of falling. | 12 weeks | N=NR | N=NR |
| Lundgren et al., 2023 | Tablet with videoconferencing software | Physiotherapist | 2x weekly 60min by videoconference | To determine whether home-based telerehabilitation could increase physical activity in CHF patients | 12 weeks | N= 31, 67.6 (±10.9) | N= 30, 67.7 (±11.9) |
| Menengiç et al., 2022 | Computer or tablet. Videoconferencing using Zoom. | Physiotherapist | 4-5x 15-40min weekly by videoconference | To investigate the effectiveness of exercise treatment via home-based telerehabilitation in Alzheimer’s disease patients. | 6 weeks | N= 10, 77.7 (±5.3) | N= 10, 80.6 (±6.1) |
| Moffet et al., 2015 | Custom videoconferencing setup | Physiotherapist | 2x 45min-1 hour weekly sessions by videoconference | To determine whether in-home telerehabilitation offered after hospital discharge to patients in the community following a total knee arthroplasty, is clinically equivalent to a face-to-face home visit approach. | 2 months | N= 104, 65 (±8) | N= 101, 67 (±8) |
| Peng et al., 2018 | Online webcam communication and telephone (app) software | Physiotherapist and nurses (cardiac and psychiatric) | 3-5x 20-30min weekly sessions by text, audio and/or video conversations | To examine the effects of a telehealth exercise training programme on health outcomes in patients with heart failure. | 2 months | N= 49, NR  Mean study age was 66.3 (±10.5) | N= 49, NR  Mean study age was 66.3 (±10.5) |
| Prvu Bettger et al., 2020 | Custom virtual telehealth system with videoconferencing setup | Physiotherapist | **Videoconference**  1x weekly, average duration not reported  Use of VERA unrestricted. | To examine costs and clinical non-inferiority of a virtual physical therapy programme compared with traditional therapy after total knee arthroplasty. | 12 weeks | N= 151, 65.4 (±7.7) | N= 153, 65.1 (±9.2) |
| Tousignant et al., 2011 | Custom videoconferencing setup | Physiotherapist | 2x 1 hour weekly sessions by videoconference | To investigate the clinical efficacy of telerehabilitation at home for patients following discharge after total knee replacement. | 8 weeks | N= 21, 66 (±10) | N= 20, 66 (±13) |
| Tsai et al., 2017 | Laptop with built-in webcam and peripherals | Physiotherapist | 3x 1 hour weekly sessions by videoconference | To examine the effectiveness of videoconferencing telerehabilitation on improving exercise capacity in patients with COPD compared with usual care. | 8 weeks | N=19, 73 (±8) | N=17, 75 (±9) |
| Wu et al., 2010 | Custom videoconferencing setup | Certified Tai Chi Chuan instructor | 3x 1 hour weekly sessions by videoconference | To examine the effectiveness of a Tai Chi tele-exercise programme among community-dwelling elders at risk for falls. | 15 weeks | N=22, 76.1 (±7.9) | N=20, 74.1 (±6.9)  N=22, 75.9 (±6.3) |
| Yerlikaya et al., 2021 | Videoconference (by e.g. WhatsApp or Google Meet). Setup details not reported | Physiotherapist | 3x 40min weekly sessions by videoconference | To investigate the effectiveness of a home-based interactive telerehabilitation programme compared with a non-supervised home exercise programme in an older population. | 8 weeks | N=18, 70.2 (±5.5) | N=16, 75.6 (±8.7)  N=16, 71.8 (±6.6) |
| Yi D. and Yim J., 2021 | Live streaming by smartphone with a-synchronous telephone contact | Physiotherapist | 2x 40min weekly sessions by live video’s. 2x weekly Individual support by telephone. | To compare the effects of a remote home-based exercise programme to improve the mental state, balance, physical function and to prevent falls in adults aged 65 years and older | 8 weeks | N=35, 76.1 (±6.3) | N=35, 77.3 (±5.6) |

^1^ = Study intervention group 1. ^2^ = Study intervention group 2.
*COPD* Chronic Obstructive Pulmonary Disorder. *CHF* Chronic Heart Failure. *THA* Total Hip Arthroplasty. *TKA* Total Knee Arthroplasty. *PD* Parkinson’s Disease. *OA* Osteoarthritis. *DM* Diabetes Mellitus.
*FP* Functional Performance. *BBS* Berg Balance Scale. *TUG* Timed-Up-And-Go test. *6MWD* 6-minute Walking Distance test. *ESWT* Endurance Shuttle Walk test. *ISWT* Incremental Shuttle Walk test. *STS* 30s sit to stand test. *10MWT* 10m walk test.

| Table 2. Description of Study Population and Factors for Generalisability of included Randomised Controlled Studies (n=26) | | | | | | | | |
| --- | --- | --- | --- | --- | --- | --- | --- | --- |
| Study | **Arm** | **Age, mean ±SD, y** | **Sampling size** | **Sex distribution** | **Baseline FP scores** | **Recruitment and retention rates for analysis** | **Recruitment location** | **Primary Medical condition** |
| Alpozgen et al., 2022 | Intervention  Control | 67.1 (±3.7  69.3 (±5.6) | N= 15  N= 15 | 5 M: 10 F  8 M: 7 F | TUG: NR  30STS: NR  TUG: NR  30STS: NR | Recruitment: N=30  Retention: 30/30 100%)  Retention IG: 15/15 (100%) | Recruitment via social media and email of older people living in urban areas | Community-dwelling older adults in isolation due to COVID-19 outbreak |
| An et al., 2021 | Intervention  Control | 71.1 (±3.3)  70.1 (±2.4)  70.4 (±2.6) | N= 18  N= 17  N= 18 | All women. | TUG: 13.84 ± 2.48  TUG: 13.27 ± 2.71  TUG: 13.01 ± 1.84 | Recruitment: N=60  Retention: 53/60 (88%)  Retention IG’s: 35/40 (88%) | Orthopaedic surgery rehabilitation hospital. | Scheduled to undergo primary TKA for treatment of knee OA |
| Bernocchi et al., 2018 | Intervention  Control | 71 (±9)  70 (±10) | N= 56  N= 56 | 50 M: 6 F  42 M: 14 F | 6MWD: 329 ± 115  6MWD: 308 ± 105 | Recruitment: N=112  Retention: 92/112 (82%)  Retention IG: 45/56 (80%) | Cardiology and pulmonary departments of rehabilitation hospitals | COPD + CHF |
| Bernocchi et al., 2019 | Intervention  Control | 77.9 (±6.0)  79.3 (±7.0) | N=141  N=142 | 57 M: 84 F  58 M: 84 F | BBS: 32.3 ± 11.6  TUG: 3.1 ± 1.2  BBS: 30.1 ± 11.8  TUG: 3.0 ± 1.1 | Recruitment: N=283  Retention: 245/283 (87%)  Retention IG: 122/141 (87%) | Rehabilitation institute | Community-dwelling older adults with fall risk and 1 or more chronic conditions*. |
| Chaplin et al., 2017 | Intervention  Control | 66 (±10)  66 (±8) | N= 51  N= 52 | 40 M: 11 F  33 M: 19 F | ISWT: 296.7 ±180.8  ESWT: 241.7 ± 209.7  ISWT: 284.2 ± 156  ESWT: 246.2 ± 144 | Recruitment: N=103  Retention: 62/103 (60%)  Retention IG: 29/51 (57%) | University hospital pulmonary rehabilitation referrals | COPD, FEV_1_ < 80% |
| Chen et al., 2017 | Intervention  Control | 66.52 (±12.08)  66.15 (±12.33) | N=27  N=27 | 18 M: 9 F  15 M: 12 F | BBS: 33.11 ± 3.98  BBS: 31.74 ± 5.89 | Recruitment: N=54  Retention: 51/54 (94%)  Retention IG: 26/27 (96%) | Shanghai 5^th^ people’s hospital (specifics not reported) | Ischaemic or haemorrhagic stroke |
| Doiron-Cadrin et al., 2020 | Intervention  Control | 69.9 (±9.1)  61.3 (±8.1)  66.7 (±9.2) | N= 11  N= 12  N= 11 | 4 M: 7 F  2 M: 10 F  3 M: 8 F | TUG: 9.4 ± 1.6  TUG: 10.7 ± 2.9  TUG: 11.4 ± 3.7 | Recruitment: N=34  Retention: 33/34 (97%)  Retention IG: 11/11 (100%) | THA or TKA waitlists of a tertiary hospital and a community hospital. | THA or TKA due to OA |
| Gandolfi et al., 2017 | Intervention  Control | 67 (±7)  70 (±9) | N= 38  N= 38 | 23 M: 15 F  28 M: 10 F | BBS: 48.63 ±6.31  10MWT: 1.59 ±.49  BBS: 45.61 ±7.97  10MWT: 1.46 ±.42 | Recruitment: N=76  Retention: 70/76 (92%)  Retention IG: 36/38 (95%) | Neurorehabilitation units | Parkinson’s Disease |
| Goode et al., 2018 | Intervention  Control | 69.6 (±3.5)  69.5 (±4.0)  71.9 (±6.5) | N= 20  N= 20  N= 20 | 19 M: 1 F  18 M: 2 F  19 M: 1 F | TUG: NR  TUG: NR  TUG: NR | Recruitment: N=60  Retention: 50/60 (83%)  Retention IG’s: 35/40 (88%) | Durham VA Health Care System | Lower Back Pain |
| Hansen et al., 2020 | Intervention  Control | 68.4 (±8.7)  68.2 (±9.4) | N= 67  N= 67 | 32 M: 35 F  28 M: 39 F | 6MWD: 322.3 ± 108.3  30STS: 9.9 ± 4.7  6MWD: 332.3 ± 97.5  30STS: 9.6 ± 3.8 | Recruitment: N=134  Retention: 100/134 (75%)  Retention IG: 57/67 (85%) | Respiratory departments of eight university hospitals | COPD, FEV_1_ < 50% |
| Holland et al., 2017 | Intervention  Control | 69 (±13)  69 (±10) | N= 80  N= 86 | 48 M: 32 F  51 M: 35 F | 6MWD: 395 ± 121  6MWD: 411 ± 107 | Recruitment: N=166  Retention: 148/166 (89%)  Retention IG: 73/80 (91%) | Tertiary hospitals’ pulmonary rehabilitation waiting lists | COPD (all severities) |
| Hong et al., 2017 | Intervention  Control | 82.2 (±5.6)  81.5 (±4.4) | N= 13  N= 13 | 5 M: 6 F  5 M: 7 F | TUG: 9.2 ± 5.7  TUG: 10.9 ± 4.8 | Recruitment: N=26  Retention: 23/26 (88%)  Retention IG: 11/13 (85%) | Senior citizen centre | Sarcopenia related factors in community-dwelling older adults |
| Hong et al., 2018 | Intervention  Control | 78.1 ± 5.66  81.54 ± 5.07 | N= 15  N= 15 | 0 M: 15 F  0 M: 15 F | TUG: 9.55 ± 4.03  TUG: 8.27 ± 2.27 | Recruitment: N=30  Retention: 23/30 (77%)  Retention IG: 10/15 (67%) | Senior citizen centre | - |
| Hwang et al., 2017 | Intervention  Control | 68 (±14)  67 (±11) | N= 24  N= 29 | 19 M: 5 F  21 M: 8 F | 6MWD: 346 ± 104  TUG: 9.4 ± 2.8  6MWD: 382 ± 106  TUG: 9.6 ± 3.7 | Recruitment: N=53  Retention: 50/53 (94%)  Retention IG: 24/24 (100%) | Cardiology and general medical wards of tertiary hospitals | Chronic heart failure |
| Li et al., 2022 | Intervention  Control | 65.3 (±8.7)  67.7 (±7.6) | N= 47  N= 48 | 35 M: 15 F  39 M: 11 F | TUG: 10.7 ± 3.5  6MWD: 445.6 ± 36.6  30STS: 11.9 ± 3.5  TUG: 10.6 ± 2.0  6MWD: 436.8 ± 43.6  30STS: 10.9 ± 3.4 | Recruitment: N=100  Retention: 95/100 (95%)  Retention IG: 47/50 (94%) | Department of cardiology of a hospital | CAD (with PCI) |
| Light et al., 2016 | Intervention  Control | NR  Mean study age: 76 | NR | 72 M: 3 F | NR | Recruitment: N=75  Retention: NR  Retention IG: NR | Outpatient clinic of a veterans administration hospital | Community-dwelling older adults with fall risk |
| Lundgren et al., 2023 | Intervention  Control | 67.6 (±10.9)  67.7 (±11.9) | N=31  N=30 | 23 M: 8 F  27 M: 3 F | 6MWD: 470 ± 123  6MWD: 461 ± 118 | Recruitment: N=61  Retention: 55/61 (90%)  Retention IG: 28/31 (90%) | Heart failure outpatient clinics | Chronic heart failure |
| Menengiç et al., 2022 | Intervention  Control | 77.7 (±5.3)  80.6 (±6.1) | N= 10  N= 10 | 3 M: 7 F  3 M: 7 F | TUG: 16.5 ± 6.9  TUG: 11.5 ± 4.1 | Recruitment: N=20  Retention: 20/20 (100%)  Retention IG: 10/10 (100%) | Outpatient clinic (screened by a neurologist) | Alzheimer’s Disease |
| Moffet et al., 2015 | Intervention  Control | 65 (±8)  67 (±8) | N= 104  N= 101 | 44 M: 60 F  56 M: 45 F | 6MWD: 324 ± 123  6MWD: 348 ± 110 | Recruitment: N=206  Retention: 198/206 (96%)  Retention IG: 98/104 (94%) | Regional hospitals’ waiting lists | Total knee arthroplasty |
| Peng et al., 2018 | Intervention  Control | NR  NR | N=49  N=49 | 28 M: 21 F  30 M: 19 F | 6MWD: 407 ± 12.3  6MWD: 406 ± 12.4 | Recruitment: N=98  Retention: 90/98 (92%)  Retention IG: 42/49 (86%) | Medical records of a teaching hospital | Chronic heart failure |
| Prvu Bettger et al., 2020 | Intervention  Control | 65.4 (±7.7)  65.1 (±9.2) | N=153  N=153 | 61 M: 90 F  30 M: 90 F | 10m gait speed: 1.0 ± 0.3**  10m gait speed: 1.0 ± 0.3** | Recruitment: N=306  Retention: N=287/306 (94%)  Retention IG: 143/153 (93%) | Academic medical centres and independent private practises | Total knee arthroplasty |
| Tousignant et al., 2011 | Intervention  Control | 66 (±10)  66 (±13) | N= 24  N= 24 | NR  NR | BBS: NR  STS: NR  TUG: NR | Recruitment: N=48  Retention: N=41/48 (85%)  Retention IG: 21/24 (88%) | Acute care hospital | Total knee arthroplasty |
| Tsai et al., 2017 | Intervention  Control | 73 (±8)  75 (±9) | N=19  N=17 | 12 M: 7 F  6 M: 11 F | 6MWD: 363 ± 66  ISWT: 260 ± 106  ESWT: 410 ± 253  6MWD: 383 ± 93  ISWT: 298 ± 114  ESWT: 361 ± 155 | Recruitment: N=37  Retention: N=36/37 (97%)  Retention IG: 19/20 (95%) | Tertiary hospital pulmonary rehabilitation programme | COPD, FEV_1_ < 80% |
| Wu et al., 2010 | Intervention  Control | 76.1 (±7.9)  74.1 (±6.9)  75.9 (±6.3) | N=22  N=20  N=22 | 3 M: 19 F  4 M: 16 F  3 M: 19 F | TUG: 13.0 ± 10.3  TUG: 8.6 ± 1.7  TUG: 9.4 ± 2.5 | Recruitment: N=64  Retention: N=51/64 (80%)  Retention IG: 20/22 (91%) | Suburban area by commercial methods | Community-dwelling older adults with fall risk |
| Yerlikaya et al., 2021 | Intervention  Control 1  Control 2 | 70.2 (±5.5)  71.8 (±6.6)  75.6 (±8.7) | N=18  N=16  N=16 | 6 M: 12 F  5 M: 11 F  4 M: 12 F | BBS: 48.1 ± 5.6  TUG: 12.4 ± 1.6  BBS: 46.3 ± 6.3  TUG: 14.9 ± 3.8  BBS: 44.2 ± 8.0  TUG: 15.8 ± 5.9 | Recruitment: N=52  Retention: N=50/52 (96%)  Retention IG: 18/18 (100%) | Randomly selected villages in Nicosia, North Cyprus | Community-dwelling older adults with fall risk |
| Yi D. and Yim J., 2021 | Intervention  Control | 76.1 (±6.3)  77.3 (±5.6) | N=35  N=35 | 8 M: 27 F  4 M: 31 F | 10m gait speed: 8.3 ± 1.3  TUG: 8.6 ± 1.4  10m gait speed: 8.0 ± 1.4  TUG: 8.2 ± 1.3 | Recruitment: N=79  Retention: N=70/79 (89%)  Retention IG: 35/38 (92%) | Senior welfare centre | Community-dwelling older adults |

* Chronic cardiac, respiratory, neurologic or neuromuscular condition(s). ** Reported as gait speed in meters per second.
*COPD* Chronic Obstructive Pulmonary Disorder. *CHF* Chronic Heart Failure. *THA* Total Hip Arthroplasty. *TKA* Total Knee Arthroplasty. *PD* Parkinson’s Disease. *OA* Osteoarthritis. *DM* Diabetes Mellitus.
*FP* Functional Performance. *BBS* Berg Balance Scale. *TUG* Timed-Up-And-Go test. *6MWD* 6-minute Walking Distance test. *ESWT* Endurance Shuttle Walk test. *ISWT* Incremental Shuttle Walk test. 30*STS* 30 seconds sit to stand test. *10MWT* 10m walk test.

| Table 3. Study Outcomes of included Randomised Controlled Studies (n=26) | | | | |
| --- | --- | --- | --- | --- |
| Study | **Intervention type** | **Control group type** | **Outcome(s)** | **Study findings** |
| Alpozgen et al., 2022 | Warm-up, strength, balance, stretching and cool-down exercises | Waitlist-control group (no care) | TUG and STS | TUG: Significant time effect (*P* = .017); Significant group by time interaction (*P* = .01) STS : Significant time effect (*P* = .001); Significant group by time interaction (*P ≤.001*)  **IG: TUG** Delta -1.2±1.8 (.0-.098)  **STS** Delta 3.3±1.4 (.0-.95)  **CG: TUG** Delta .6±1.0 (.0-.095)  **STS** Delta .5±1.3 (.033-.3) |
| An et al., 2021 | Warm-up, mobility, flexibility, strength, balance, and cool-down exercises | Usual care | TUG for dynamic balance. | Significant differences between groups (*F[2,50]=3.584, P=0.035,ⴄ^2^_p_ = .125)* and group-by-time interaction for quadricep strength and TUG time (*F[4,100]=7.252, P* < .001*,ⴄ^2^_p_ = .225).*  **IG1: TUG** pre-test: 13.8±2.5 post-test: 10.6±1.3  **IG2: TUG** pre-test: 13.3±2.7 post-test: 12.2±1.6  **CG: TUG** pre-test: 13.0±1.8 post-test: 13.3±2.1 |
| Bernocchi et al., 2018 | Telerehabilitation exercise training | Conventional care via GP, hospital check-ups on demand and an educational session on a healthy lifestyle | Exercise tolerance improvement measured by the difference walked at the 6MWD. | Significant difference between groups in favour of the IG for difference in distance walked at the 6MWD (*P* = .004). and a significant group by time interaction (*P* = .0001, more details NR).*  **IG: 6MWD** T0-T1 delta: 60m (95% CI 22.2-97.8) T1-T2 delta 7m (95% CI -11.6-25.7)  **CG: 6MWD** T0-T1 delta: -15m (95% CI -40.3-9.8) T1-T2 delta 7m (95% CI -63.5-22.2) |
| Bernocchi et al., 2019 | Home-based exercise training with weekly telephone and monthly videoconference telerehabilitation guidance | Conventional care via GP + standard fall risk advice. | Fall risk and gait + balance measure by BBS and TUG. | Significant group by time interaction in favour of the IG for the TUG and BBS (*P < .001, details NR).*  **IG: TUG** pre-test: 3.1 (95% CI 2.9-3.3) post-test: 2.5 (95% CI 2.3-2.7)  **BBS** pre-test: 32.3 (95% CI 30.4-34.3) post-test: 38.5 (95% CI 36.4-40.7)  **CG: TUG** pre-test: 3.0 (95% CI 2.9-3.2) post-test: 2.8 (95% CI 2.6-3.1)  **BBS** pre-test: 30.1 (95 CI 28.2-32.1) post-test: 32.8 (95% CI 30.3-35.3) |
| Chaplin et al., 2017 | Web-based pulmonary rehabilitation | Conventional pulmonary rehabilitation, either hospital or community based. | Exercise capacity measured by the ESWT and ISWT. | ESWT: Significant time effect (*P* < .01) for IG and CG; No significant between-group difference (*P* NR). ISWT: No significant time effect (*P* NR); No significant between-group difference (*P* NR).  Interaction effect (group by time) NR.  **IG: ESWT** pre-test: 241.7±209.7 post-test: MD 189.0±211.1  **ISWT** pre-test: 296.7±180.8 post-test: NR, MCID 48m  **CG: ESWT** pre-test: 246.2±144.0 post-test: MD 184.5±247.4  **ISWT** pre-test: 284.2±156.0 post-test: NR, MCID: 48m |
| Chen et al., 2017 | Telerehabilitation with integrated ETNS exercise training | Conventional outpatient/centre-based rehabilitation. | Balance change was measured by BBS. | Significant time effect (*P* < .001); No significant group effect (F=0.012, *P =*.247); No significant group by time interaction (F=1.423, *P* = .912).  **IG: BBS** pre-test: 33.11±3.98 post-test: 40.46±4.12  **CG: BBS** pre-test: 31.74±5.89 post-test: 39.50±5.41 |
| Doiron-Cadrin et al., 2020 | Multi-component rehabilitation programme | 2 control groups; in-person prehabilitation sessions and usual care before THA/TKA. | Mobility, balance and fall risk by the TUG. | No significant time effect (*P* NR); No significant group by time interaction (*P* = .282).  **IG: TUG** pre-test: 9.4±1.6 post-test: 8.5±1.4 (MD -0.8±1.7, 95% CI -1.9-0.3)  **CG1: TUG** pre-test: 10.7±2.9 post-test: 10.6±2.9 (MD -0.2±1.7, 95% CI -1.3-1.0)  **CG2: TUG** pre-test: 11.4±3.7 post-test: 11.8±4.4 (MD 0.3±1.5, 95% CI -0.7-1.4) |
| Gandolfi et al., 2017 | Virtual reality duo-based TeleWii training | Sensory integration balance training performed in-clinic. | Static and dynamic balance change measured by BBS and 10MWT. | BBS: Significant time effect (*P* < .001); Significant group difference (*P* = .04); No significant group-time effect (*P* = NR). 10MWT: Significant time effect (*P =* .02); No significant group difference or group-time effect (*P* = NR).  **IG: BBS** pre-test: 48.63±6.31 post-test: 52.37±3.29  **10MWT** pre-test: 1.59±0.49 post-test: 1.62± 0.43  **CG: BBS** pre-test: 45.61±7.97 post-test: 49.82±5.70  **10MWT** pre-test: 1.46±0.42 post-test: 1.60±0.44 |
| Goode et al., 2018 | Physical activity (+ cognitive-behavioural therapy) with weekly telephone guidance | Waitlist-control group (no care) | TUG | Small to medium treatment effects were found for both intervention groups compared to the control group based on Cohen *d .28-.31.*  **IG1: TUG** MD -2.94, 95% CI -6.24-0.35, effect size -.28  **IG2: TUG** MD -3.26, 95% CI -6.69-0.18, effect size -.31  **CG: TUG** MD 1.11, 95% CI -1.25-3.47 |
| Hansen et al., 2020 | Pulmonary group-based telerehabilitation | Conventional centre-based rehabilitation | Exercise capacity change measured by 6MWD and STS test. | 6MWD: Significant time effect (*P* <.05); No significant group by time interaction (*P* NR). STS: Significant time effect (*P* <.05); No significant group by time interaction (*P* NR). No improvement exceeded the MCID.  **IG: 6MWD** pre-test 322.3±108.3 post-test MD 17.2 (95% CI 5.8-28.5)  **30STS** pre-test 9.9±4.7 post-test MD 1.3 (95% CI 0.4-2.0)  **CG: 6MWD** pre-test 332.3±97.5 post-test MD 23.5 (95% CI 12.1-35.0)  **30STS** pre-test 9.6±3.8 post-test MD 1.7 (95% CI 0.9-2.5) |
| Holland et al., 2017 | Home-based pulmonary rehabilitation with weekly telephone guidance | Conventional centre-based rehabilitation | Exercise capacity change measured by 6MWD | No significant between-group difference after 8 weeks in 6MWD (*P* NR). Non-inferiority was established, superiority could not be excluded. Between group difference of 18.57m (95% CI -3.32 – 40.71, *P* NR).  **IG: 6MWD** pre-test 395±121 post-test MD 29.4 (95% CI 13.8-45.0)  **CG: 6MWD** pre-test 411±107 post-test MD 10.8 (95% CI -4.5-26.2) |
| Hong et al., 2017 | Telerehabilitation exercise training | Nutrition and exercise education every 4 weeks, checked by phone every 2 weeks | Functional fitness measured by the TUG and STS test. | TUG: No significant time effect (*P* = .183); No significant group by time interaction (*P* = .956). STS: Significant time effect (*P* = .035); Significant between-group difference (*P* = .017); No significant group by time interaction (*P* = .158).  **IG: TUG** pre-test: 9.2±5.7 post-test: 8.1±2.2  **STS** pre-test: 12.3±2.8 post-test: 15± 3.9  **CG: TUG** pre-test: 10.9±4.8 post-test: 9.7±5.0  **STS** pre-test: 9.2±4.1 post-test: 9.8±5.5 |
| Hong et al., 2018 | Telerehabilitation exercise training | Nutrition and exercise education every 4 weeks, checked by phone every 2 weeks | Fall-related risk factors by STS and BBS test. | BBS: Significant group by time interaction (*P* = .03). TUG: No significant time effect (*P* NR); No significant group by time interaction (*P* = .40). STS: Significant group by time interaction (*P* < .001).  **IG: TUG** pre-test: 9.6±4.0 post-test: 8.9±2.8  **BBS** pre-test: 43.0±6.5 post-test: 44.3±6.3  **STS** pre-test: 11.0±4.6 post-test: 19.2±6.0  **CG: TUG** pre-test: 8.3±2.3 post-test: 8.5±1.8.0  **BBS** pre-test: 44.7±3.5 post-test: 43.8±3.6  **STS** pre-test: 13.0±2.6 post-test: 14.2± 2.7 |
| Hwang et al., 2017 | Group-based telerehabilitation exercise training | Conventional centre-based rehabilitation | Non-inferiority by 6MWD, TUG and 10m gait speed test. | 6MWD: Significant time effect (F[2,6]=3.23; *P* = .048); No significant group by time interaction (F[1,6]=1.39; *P* = .24). TUG: No significant time effect (*P* NR); No significant group by time interaction (*P* NR). 10MWT: No significant time effect (*P* NR); No significant group by time interaction (*P* NR).  **IG: 6MWD** pre-test 346±104 post-test: 364±96  **TUG** pre-test: 9.4±2.8 post-test: 8.9±3.0  **10 MWT** pre-test: 7.2±1.8 post-test: 7.1±2.4  **CG: 6MWD** pre-test 382±106 post-test: 394±119  **TUG** pre-test: 9.6±3.7 post-test: 9.7±5.4  **10 MWT** pre-test: 7.4±2.5 post-test: 7.4±3.0 |
| Li et al., 2022 | Home-based online supervised exercise programme | Conventional in-person health education and a home exercise programme | TUG, STS and 6MWD | STS: Significant group by time interaction (*P* < .001, 95% CI 1.4-3.4). 6MWD: Significant time effect (*P* < .001, 95% CI 32.7-54.7), significant group by time interaction (*P* = .001, 95% CI 11.5-42.6). TUG: Significant time effect (*P* = .001, 95% CI 0.3-1.2).  **IG: 6MWD** pre-test 445.6±36.6 post-test: 489.2±48.8  **TUG** pre-test: 10.7±3.5 post-test: 9.9±3.0  **STS** pre-test: 11.9±3.5 post-test: 14.3±4.7  **CG: 6MWD** pre-test 436.8±43.6 post-test: 453.4±50.7  **TUG** pre-test: 10.6±2.0 post-test: 9.7±1.9  **STS** pre-test: 10.9±3.4 post-test: 11.4±3.5 |
| Light et al., 2016 | Home-based balance training with weekly telephone guidance | Home-based balance training without guidance | BBS | Significant effect over time (*P* < .001), and a significant group by time interaction(*P* = .039).  No exact data of improvements reported. |
| Lundgren et al., 2023 | Group-based telerehabilitation exercise training | Usual care by advise to follow current guidelines for CHF | 6MWD | Significant time effect (delta 19.1m , 95% CI 3.0-35.1, *P* = .02). No significant difference between groups (delta 3.84m, 95% CI -18.88-26.55, *P* = .741)  **IG: 6MWD** pre-test: 470±123 post-test: 483.71 (95% CI 452.43-515.0)  **CG: 6MWD** pre-test: 461±118 post-test: 479.88 (95% CI 448.44-511.32 |
| Menengiç et al., 2022 | Real-time exercise treatment with supervision by videoconference | Usual daily activities | TUG | No significant between group difference (*P* =.248)*.* Significant group by time interaction in favour of the IG for mean TUG change. (*P* = .002, Mean differences IG: -3.83 ± 0.97 (−8.14 to 2.4) CG: 2.22 ± 0.93 (−1.77 to 8.28)).  **IG: TUG** pre-test: 16.5±6.9 post-test: 12.7±6.8  **CG: TUG** pre-test: 11.5±4.1 post-test: 13.8±3.3 |
| Moffet et al., 2015 | Telerehabilitation exercise training | Conventional home visit rehabilitation | 6MWD | No significant group by time interaction (*P* NR). Non-inferiority confirmed. PPanalysis MD -15.5m (95% CI -35.6-4.5). ITTanalysis MD -7.6m (95% CI -27.0-11.9).  **IG: 6MWD** pre-test: 324±123 post-test: 382.1±6.4  **CG: 6MWD** pre-test: 348±110 post-test: 363.6±5.9 |
| Peng et al., 2018 | Telerehabilitation exercise training | Usual care consisting of general advice and regular clinical follow-ups | Exercise capacity measured by the 6MWD. | Significant effect over time (Fw=37.19, *P* < .001) and for group by time interaction (Fin=73.09, *P* < .001).  **IG: 6MWD** pre-test: 407.09±12.27 post-test: 419.23±9.67  **CG: 6MWD** pre-test: 406.05±12.35 post-test: 406.55±12.54 |
| Prvu Bettger et al., 2020 | Virtual exercise training system + telerehabilitation videoconference | Conventional home visit/outpatient rehabilitation | Clinical effectiveness by the 10MWT. | No significant time effect (*P* NR); No significant group by time interaction (*P* = .199). MD -0.04m/s (95% CI -.10-0.01). Non-inferiority confirmed.  **IG: 10MWT** pre-test: 1.0±0.3 post-test: 1.0± 0.3  **CG: 10MWT** pre-test: 1.0±0.3 post-test: 1.0±0.3 |
| Tousignant et al., 2011 | Telerehabilitation exercise training | Conventional home visit/outpatient rehabilitation | BBS, STS and TUG test. | BBS: Significant time effect (*P* NR); No significant group by time interaction (*P* NR). TUG: Significant time effect (*P* NR); No significant group by time interaction (*P* NR). STS: Significant time effect (*P* NR); No significant group by time interaction (*P* NR).  No exact data of improvements reported. |
| Tsai et al., 2017 | Group-based telerehabilitation exercise training | Usual medical management without participation in exercise training. | Exercise capacity measured by the ESWT, ISWT and 6MWD. | ESWT: Significant time effect (*P* NR) and significant group by time interaction (*P* < .001). ISWT: No significant time effect (*P* NR); No significant group by time interaction (*P* = .66). 6MWD: Significant time effect (*P* NR); No significant group by time interaction (*P* = .16).  **IG: ESWT** pre-test: 410±253 post-test: 693±357, MD 283 (95%CI 107-460)  **ISWT** pre-test: 260±106 post-test: 275±132, MD 12 (95% CI -12-36)  **6MWD** pre-test: 363±66 post-test: 403±82, MD 40 (95% CI 1-80)  **CG: ESWT** pre-test: 361±155 post-test: 316±182, MD -31 (95% CI -76-14)  **ISWT** pre-test: 298±114 post-test: 306±118, MD 8 (95% CI -8-24)  **6MWD** pre-test: 383±93 post-test: 374±136, MD -9 (95% CI -62-44) |
| Wu et al., 2010 | Group-based Tai-chi tele-exercise | 2 control groups; traditional community centre-based and home video-based | Fall risk by TUG | No significant time effect (*P* = .60); No significant between-group difference (*P* = .80).  IG: TUG pre-test: 13.0±10.3 post-test: -0.8±6.7  **CG1: TUG** pre-test: 8.6±1.7 post-test: -0.2±1.2  **CG2: TUG** pre-test: 9.4±2.5 post-test: 0.0±0.8 |
| Yerlikaya et al., 2021 | Group based balance and strengthening exercises | 2 control groups; non-supervised home exercise with video and daily routine. | Balance, mobility and postural sway by TUG and BBS. | Significant time effect for the IG and the non-supervised home exercise group (CG1) for both the TUG and BBS (*P* <.001).  **IG: TUG** pre-test: 12.38±1.59 post-test: 8.98±2.35  **BBS** pre-test: 48.11±5.66 post-test: 53.33±4.22  **CG1: TUG** pre-test: 14.88±3.80 post-test: 10.61±3.47  **BBS** pre-test: 46.31±6.32 post-test: 52.12±5.28  **CG2: TUG** pre-test: 15.82±5.88 post-test: 15.92±5.73  **BBS**  pre-test: 44.25±8.02 post-test: 46.87±8.67 |
| Yi D. and Yim J. 2021 | A programme of stretches, vestibular rehabilitation, core and limb strengthening, joint range of motion and balance exercises. | Usual daily activities | Balance and mobility by TUG and 10MWT. | 10MWT: Significant time effect (*P* NR). Significant group by time interaction (*F[1,68]=9.612, P=0.003).* TUG: Significant time effect (*P* NR). Significant group by time interaction (F[1,68]=7.289, P=0.009).  **IG: TUG** pre-test: 8.60±1.42 post-test: 7.71±1.31  **10 MWT** pre-test: 8.33±1.28 post-test: 7.50±1.30  **CG: TUG** pre-test: 8.15±1.33 post-test: 7.83±1.30  **10 MWT** pre-test: 8.00±1.38 post-test: 7.81±1.30 |

*NR* Not Reported. * Data found in supplementary files. *MD* Mean Difference. *95%CI* 95% confidence interval. *IG1/IG2/CG1/CG2* Intervention group 1-2, Control group 1-2.
*COPD* Chronic Obstructive Pulmonary Disorder. *CHF* Chronic Heart Failure. *THA* Total Hip Arthroplasty. *TKA* Total Knee Arthroplasty. *PD* Parkinson’s Disease. *OA* Osteoarthritis. *DM* Diabetes Mellitus. *ETNS* Electromyography-triggered neuromuscular stimulation.
*FP* Functional Performance. *BBS* Berg Balance Scale. *TUG* Timed-Up-And-Go test. *6MWD* 6-minute Walking Distance test. *ESWT* Endurance Shuttle Walk test. *ISWT* Incremental Shuttle Walk test. *STS* 30s sit to stand test. *10MWT* 10m walk test. *IG* Intervention Group. *CG* Control Group.

**Table 4. Revised risk-of-bias assessment of included Randomised Controlled Studies (n=26)**

| **Study** | **Overall risk of bias** | **Domain 1: The randomisation process** | **Domain 2: Deviations from the intended interventions** | **Domain 3: Missing outcome data** | **Domain 4: Measurement of the outcome** | **Domain 5: Selection of the reported results** | **Other sources of bias** |
| --- | --- | --- | --- | --- | --- | --- | --- |
| **Alpozgen et al., 2022** | **Some concerns** | 1.1 + 1.2 probably yes. The method of concealment is not fully described. Insufficient information about the randomisation process is given(e-picos website). No problems with baseline group differences. | 2.1 + 2.2 No. 2.6 NI. 2.7 No. | 3.1 Yes | 4.1 + 4.2 + 4.3 No. | 5.2 + 5.3 No. 5.1 Yes but insufficient info present in the protocol to permit judgement. | - |
| **An et al., 2021** | **Some concerns** | Participants were randomised using a computerized random number generator. 1.2 PY: Allocation was concealed by use sealed envelopes. No problems with baseline group differences. | 2.1 + 2.2 Yes. However, no deviations from the intended intervention arose due to the trial context. 2.6 NI, 2.7 PN. | 3.1 Yes. Missing outcome data was balanced across groups with no important difference to the estimated effect of intervention. Each main outcome was reported with complete data. | 4.1 + 4.2 + 4.3 No. | 5.2 + 5.3 No. 5.1 The protocol of the trial was added retrospectively. | - |
| **Bernocchi et al., 2018** | **High risk** | Randomisation using computer generated random allocation sequence of blocks of 4. Allocation was concealed by use of sequentially numbered opaque sealed envelopes. No problems with baseline group differences. | 2.1 + 2.2 Yes. However, no deviations from the intended intervention arose due to the trial context. 2.6 PY. ITT analyses was used. | T1 timepoint (4 months): 8/56 missing from intervention group and 12/56 from control group. Reasons differ across groups. 3.2 NI. 3.3 + 3.4 PY. | 4.1 + 4.2 + 4.3 No. | 5.2 + 5.3 No. 5.1 Yes. The study protocol is available and the published report includes all expected outcomes. | Unclear. Due to attrition the CG was left with fewer minimum numbers calculated for sample size. |
| **Bernocchi et al., 2019** | **Low risk** | 1.1 + 1.2 probably yes. The method of concealment is not fully described. No problems with baseline imbalances. | 2.1 + 2.2 Yes. However, no deviations from the intended intervention arose due to the trial context. 2.6 PY. ITT analyses was used. | 3.1 Yes. Missing outcome data was balanced across groups with no important difference to the estimated effect of intervention. Each main outcome was reported with complete data. | 4.1 + 4.2 + 4.3 No. | 5.2 + 5.3 No. 5.1 Yes. The study protocol is available and the published report includes all expected outcomes. | - |
| **Chaplin et al., 2017** | **High risk** | 1.1 + 1.2 probably yes. The method of concealment is not fully described. Insufficient information about the sequence generation process is given. No problems with baseline group differences. | 2.1 + 2.2 Yes. However, no deviations from the intended intervention arose due to the trial context. 2.6 NI, 2.7 PN. | 8 weeks: 29/51 missing from intervention group and 12/52 from control group. Reasons differ across groups. 3.2 NI. 3.3 + 3.4 PY. | 4.1 + 4.2 + 4.3 No. | 5.2 + 5.3 No. 5.1 Yes. The study protocol is available and the published report includes all expected outcomes. | - |
| **Chen et al., 2017** | **Some concerns** | Participants were randomised using a computer-generated blocked randomization sequence. Allocation was concealed by use of opaque sealed envelopes. No problems with baseline group differences. | 2.1 + 2.2 Yes. However, no deviations from the intended intervention arose due to the trial context. 2.6 PY. ITT analyses was used. | 3.1 Yes. Missing outcome data was balanced across groups with no important difference to the estimated effect of intervention. Each main outcome was reported with complete data. | 4.1 + 4.2 + 4.3 No. | 5.2 + 5.3 No. 5.1 Yes but discrepancies exist between final article and study protocol. | - |
| **Doiron-Cadrin et al., 2020** | **Low risk** | Participants were randomised using a random number generator. Allocation was concealed by use of opaque sealed envelopes. No problems with baseline group differences. | 2.1 + 2.2 Yes. However, no deviations from the intended intervention arose due to the trial context. 2.6 PY. ITT analyses was used. | 3.1 Yes | 4.1 + 4.2 + 4.3 No. | 5.2 + 5.3 No. 5.1 Yes. The study protocol is available and the published report includes all expected outcomes. | - |
| **Gandolfi et al., 2017** | **Some concerns** | 1.2 NI, 1.3 N. The method of concealment is not described. Participants were randomised using computer generated random number tables. No problems with baseline imbalances. | 2.1 + 2.2 Yes. However, no deviations from the intended intervention arose due to the trial context. 2.6 NI, 2.7 PN. | 3.1 Yes. Missing outcome data was balanced across groups with no important difference to the estimated effect of intervention. Each main outcome was reported with complete data. | 4.1 + 4.2 + 4.3 No. | 5.2 + 5.3 No. 5.1 NI. Insufficient information to permit judgement. | - |
| **Goode et al., (2018)** | **Some concerns** | 1.1 Yes, 1.2 PY. The method of allocation sequence concealment is not fully described. No problems with baseline group differences. | 2.1 + 2.2 Yes. However, no deviations from the intended intervention arose due to the trial context. 2.6 PY. | 3.1 No. However, missing outcome data was balanced across groups but with no data on reasons of withdrawal. 3.4 PN. | 4.1 + 4.2 + 4.3 No. | 5.2 + 5.3 No. 5.1 Yes. The study protocol is available and the published report includes all expected outcomes. |  |
| **Hansen et al., 2020** | **Low risk** | 1.1 + 1.2 probably yes. The method of concealment is not fully described. Participants were randomised using a computer-generated randomisation list. No problems with baseline group differences. | 2.1 + 2.2 Yes. However, no deviations from the intended intervention arose due to the trial context. 2.6 PY. ITT analyses was used. | Reasons for missing outcome data unlikely to be related to true outcome with no important difference to the estimated effect of intervention. | 4.1 + 4.2 + 4.3 No. | 5.2 + 5.3 No. 5.1 Yes. The study protocol is available and the published report includes all expected outcomes. | Unclear. Variations in exercise content and volume exist in the conventional control PR group, though it is not specified what these variations are. |
| **Holland et al., 2017** | **Low risk** | Allocation was concealed by use of opaque sealed envelopes. Participants were randomised using a computer-generated sequence. No problems with baseline group differences. | 2.1 + 2.2 Yes. However, no deviations from the intended intervention arose due to the trial context. 2.6 PY. ITT analyses was used. | 3.1 Yes. Missing outcome data was balanced across groups with no important difference to the estimated effect of intervention. Each main outcome was reported with complete data. | 4.1 + 4.2 + 4.3 No. | 5.2 + 5.3 No. 5.1 Yes. The study protocol is available and the published report includes all expected outcomes. | - |
| **Hong et al., 2017** | **Some concerns** | 1.2 NI, 1.3 N. The method of concealment is not described. Insufficient information about the sequence generation process is given. No problems with baseline imbalances. | 2.1 + 2.2 Yes. However, no deviations from the intended intervention arose due to the trial context. 2.6 NI, 2.7 PN. | 3.1 Yes | 4.1 + 4.2 No. 4.3 NI. The study did not address this outcome. 4.4 Y 4.5 PN. | 5.2 + 5.3 No. 5.1 NI. Insufficient information to permit judgement. | - |
| **Hong et al., 2018** | **High risk** | Group allocation was unconcealed. Random sampling was performed in Microsoft Excel, though insufficient information about the sequence generation process is given. | 2.1 + 2.2 Yes. However, no deviations from the intended intervention arose due to the trial context. 2.6 NI, 2.7 PN. | 12 weeks: 5/15 missing from intervention group and 2/15 from control group. Reasons differ across groups. 3.2 NI. 3.3 + 3.4 PY. | 4.1 + 4.2 + 4.3 No. | 5.2 + 5.3 No. 5.1 NI. Insufficient information to permit judgement. Protocol found yet no statistical analysis plan was included. | - |
| **Hwang et al., 2017** | **Some concerns** | Allocation was concealed by use of opaque, sealed and numbered envelopes. Participants were randomised using a non-blocked random allocation sequence. No problems with baseline group differences. | 2.1 + 2.2 Yes. However, no deviations from the intended intervention arose due to the trial context. 2.6 PY. ITT analyses was used. | 3.1 Yes. Missing outcome data was balanced across groups with no important difference to the estimated effect of intervention. Each main outcome was reported with complete data. | 4.1 + 4.2 + 4.3 No. | 5.2 + 5.3 No. 5.1 NI. Insufficient information to permit judgement. | Unclear. The extent to which participants carried out home exercises beyond the formal sessions was not evaluated. No *P* values were reported for secondary outcomes. |
| **Li et al., 2022** | **Some concerns** | Randomly allocated, with no further information.  1.1 Yes. 1.2 NI. 1.3 No. | 2.1 No. 2.2 NI. 2.3 PN. | 3.1 Yes. Missing outcome data was balanced across groups with no important difference to the estimated effect of intervention. Each main outcome was reported with complete data. | 4.1 N. 4.2 N. 4.3 NI. 4.4 PN. 4.5 PN | 5.2 + 5.3 No. 5.1 Yes. The study protocol is available and the published report includes all expected outcomes. | - |
| **Light et al., 2016** | **High risk** | Initial randomisation by coin toss, continued by pair randomization. NI on concealments. NI on baseline imbalances. | 2.1 + 2.2 NI. The assessor was blinded. 2.6 NI + 2.7 PN. | No participant data available other than the primary outcome results. | 4.1 + 4.2 + 4.3 No. | 5.2 + 5.3 No. 5.1 NI. Insufficient information to permit judgement. | The article is lacking information on several domains. |
| **Lundgren et al., 2023** | **Low risk** | 1.1 Yes, 1.2 PY. The method of allocation sequence concealment is not fully described. No problems with baseline group differences. | 2.1 + 2.2 Yes. However, no deviations from the intended intervention arose due to the trial context. 2.6 PY. ITT analyses was used. | 3.1 Yes. Missing outcome data was balanced across groups with no important difference to the estimated effect of intervention. Each main outcome was reported with complete data. | 4.1 + 4.2 + 4.3 No. | 5.2 + 5.3 No. 5.1 Yes. The study protocol is available and the published report includes all expected outcomes. | - |
| **Menengiç et al., 2022** | **High risk** | Participants were stratified by sex and baseline dementia severity, then randomized to one of the two groups. Participants were asked to choose a number and assigned into two groups in a 1:1 ratio according to the random numbers generated using web-based software. No problems with baseline group differences. NI on concealments. | 2.1 + 2.2 Yes. However, no deviations from the intended intervention arose due to the trial context. 2.6 NI, 2.7 PN. | 3.1 Yes. Missing outcome data was balanced across groups with no important difference to the estimated effect of intervention. Each main outcome was reported with complete data. | 4.1 PN. No ANOVA was used. 4.2 N. 4.3 PY. 4.4 PY. 4.5 PY | 5.2 + 5.3 No. 5.1 Yes. The study protocol is available and the published report includes all expected outcomes. | - |
| **Moffet et al., 2015** | **Some concerns** | There was a concealed allocation to one of the two study groups. Participants were randomised using a computer-generated sequence. No problems with baseline group differences. | 2.1 + 2.2 Yes. However, no deviations from the intended intervention arose due to the trial context. ITT analyses was used secondarily. | 3.1 Yes. Missing outcome data was balanced across groups with no important difference to the estimated effect of intervention. Each main outcome was reported with complete data. | 4.1 + 4.2 + 4.3 No. | 5.2 + 5.3 No. 5.1 NI. Insufficient information to permit judgement. Protocol found yet no statistical analysis plan was included. | Intervention duration was 2 months; however, outcomes were assessed at 4 months post-discharge. |
| **Peng et al., 2018** | **High risk** | Allocation was concealed by use of opaque sealed envelopes. Participants were randomised using a simple computer-generated sequence. No problems with baseline group differences. | 2.1 + 2.2 Yes. However, no deviations from the intended intervention arose due to the trial context. 2.6 NI, 2.7 PN. | 8 weeks: 7/49 missing from intervention group and 8/49 from control group. Reasons differ across groups. 3.2 NI. 3.3 + 3.4 PY. | 4.1 + 4.2 + 4.3 No. | 5.2 + 5.3 No. 5.1 NI. Insufficient information to permit judgement. | - |
| **Prvu Bettger et al., 2020** | **Some concerns** | 1.2 NI, 1.3 N. The method of concealment is not described. Participants were randomised although it is not specified how. No problems with baseline imbalances. | 2.1 + 2.2 Yes. However, no deviations from the intended intervention arose due to the trial context. 2.6 PY. ITT analyses was used. | 3.1 Yes. Missing outcome data was balanced across groups with no important difference to the estimated effect of intervention. Each main outcome was reported with complete data. | 4.1 + 4.2 No. 4.3 NI. The study did not address this outcome. 4.4 Yes. 4.5 PN | 5.2 + 5.3 No. 5.1 Yes. The study protocol is available and the published report includes all expected outcomes. | - |
| **Tousignant et al., 2011** | **High risk** | Allocation to study groups was done using sealed envelopes. Participants were randomised using a random number generator. No problems with baseline group differences. | 2.1 + 2.2 Yes. However, no deviations from the intended intervention arose due to the trial context. 2.6 NI, 2.7 PN. | Exclusion was reported though individual outcome data were absent. 3.2 NI. 3.3 + 3.4 PY. | 4.1 + 4.2 No. 4.3 PN. The study did not specifically address blinding. | 5.2 + 5.3 No. 5.1 NI. Insufficient information to permit judgement. | Possible selection bias due to dropout of participants who were unhappy with their designated group. |
| **Tsai et al., 2017** | **Some concerns** | There was a concealed allocation to one of the two study groups. Participants were randomised using a computer-generated sequence. No problems with baseline group differences. | 2.1 + 2.2 Yes. However, no deviations from the intended intervention arose due to the trial context. 2.6 PY. ITT analyses was used. | 3.1 Yes. Missing outcome data was balanced across groups with no important difference to the estimated effect of intervention. Each main outcome was reported with complete data. | 4.1 + 4.2 + 4.3 No. | 5.2 + 5.3 No. 5.1 NI. Insufficient information to permit judgement. Protocol found yet no statistical analysis plan was included. | Control group did not participate in exercise training, though it was not specified whether this was specifically disallowed or prohibited or simply advised against doing. |
| **Wu et al., 2010** | **High risk** | Allocation concealment was not described. Participants were randomised although it is not specified how. No problems with baseline imbalances. | 2.1 + 2.2 Yes. However, no deviations from the intended intervention arose due to the trial context. 2.6 PY. ITT analyses was used. | 15 weeks: 2/22 missing from intervention group and 11/44 from control groups. Reasons differ across groups. 3.2 NI. 3.3 + 3.4 PY. | 4.1 + 4.2 No. 4.3 NI. The study did not address this. 4.4 + 4.5 PY. | 5.2 + 5.3 No. 5.1 NI. Insufficient information to permit judgement. | - |
| **Yerlikaya et al., 2021** | **Some concerns** | Participants were randomised using random blocks of six. Allocation was concealed by use of sealed envelopes. Randomization was stratified by sex. No problems with baseline group differences. | 2.1 + 2.2 Yes. However, no deviations from the intended intervention arose due to the trial context. 2.6 NI, 2.7 PN. | 3.1 Yes. Missing outcome data was balanced across groups with no important difference to the estimated effect of intervention. Each main outcome was reported with complete data. | 4.1 + 4.2 + 4.3 No. | 5.2 + 5.3 No. 5.1 NI. Insufficient information to permit judgement. | - |
| **Yi D. and Yim J., 2021** | **High risk** | Initial randomisation by random number generator. NI on concealments. NI on baseline imbalances. | 2.1 + 2.2 NI. 2.3 PN. 2.6 NI. 2.7 PN | 3.1 Yes. Missing outcome data was balanced across groups with no important difference to the estimated effect of intervention. Each main outcome was reported with complete data. | 4.1 + 4.2 No. 4.3 NI. The study did not address this. 4.4 + 4.5 PY. | 5.2 + 5.3 No. 5.1 NI. Insufficient information to permit judgement. | - |
